# Trichostatin A enhances the titanium rods osseointegration in osteoporotic rats by the inhibition of oxidative stress through activating the AKT/Nrf2 pathway

**DOI:** 10.1038/s41598-023-50108-1

**Published:** 2023-12-27

**Authors:** Zhi Zhou, Wenkai Jiang, Junjie Yan, Hedong Liu, Maoxian Ren, Yang Li, Zhiyi Liu, Xuewei Yao, Tianlin Li, Nengfeng Ma, Bing Chen, Wengang Guan, Min Yang

**Affiliations:** Department of Traumatology and Orthopedics, Yijishan Hospital, Wannan Medical College, Wuhu, 241001 Anhui People’s Republic of China

**Keywords:** Stem cells, Zoology, Diseases, Medical research, Pathogenesis

## Abstract

The use of titanium implants as fixed supports following fractures in patients with OP can often result in sterile loosening and poor osseointegration. Oxidative stress has been shown to play a particularly important role in this process. While TSA has been reported to facilitate in vivo osteogenesis, the underlying mechanisms remain to be clarified. It also remains unclear whether TSA can improve the osseointegration of titanium implants. This study investigated whether TSA could enhance the osseointegration of titanium rods by activating AKT/Nrf2 pathway signaling, thereby suppressing oxidative stress. MC3T3-E1 cells treated with CCCP to induce oxidative stress served as an in vitro model, while an OVX-induced OP rat model was employed for in vivo analysis of titanium rod implantation. In vitro, TSA treatment of CCCP-treated MC3T3-E1 cells resulted in the upregulation of osteogenic proteins together with increased AKT, total Nrf2, nuclear Nrf2, HO-1, and NQO1 expression, enhanced mitochondrial functionality, and decreased oxidative damage. Notably, the PI3K/AKT inhibitor LY294002 reversed these effects. In vivo, TSA effectively enhanced the microstructural characteristics of distal femur trabecular bone, increased BMSCs mineralization capacity, promoted bone formation, and improved the binding of titanium implants to the surrounding tissue. Finally, our results showed that TSA could reverse oxidative stress-induced cell damage while promoting bone healing and improving titanium rods' osseointegration through AKT/Nrf2 pathway activation.

## Introduction

Rates of Osteoporosis (OP)-related fractures represent an increasingly pressing issue with the aging of the global population^1,2^. In prior reports, oxidative stress has been shown to influence OP onset and progression^3^. Oxidative stress occurs in cells when reactive oxygen species (ROS) generation surpasses the antioxidant system's capacity to neutralize these harmful free radicals^4–6^. This process can drive the OP-related disruption of bone homeostatic remodeling, subsequently leading to a decreased bone mineral density (BMD)^7^. The mitochondria are the primary site of ROS generation in eukaryotic cells^8^, and excessive ROS levels can diminish the mitochondrial membrane potential (MMP), impair mitochondrial oxidative phosphorylation, and ultimately contribute to apoptotic cell death^9^. Mitigating oxidative stress is thus central to protecting osteoblast integrity and preventing OP^10^. In prior studies, the AKT/Nrf2 signaling pathway has been identified as a key mediator of antioxidant activity^11^, with its activation ultimately contributing to reductions in intracellular ROS levels^12^. There is, thus, an imperative need for further studies to probe the link between OP and the regulatory role of AKT/Nrf2 signaling.

Given the limited efficacy of medications for OP-related fractures, orthopedic internal fixation surgery has become as a primary therapeutic strategy for affected patients^13^. Titanium and its alloys are favored in the design of orthopedic implants due to their excellent biocompatibility and robust physicochemical stability^14^. While outcomes associated with orthopedic implants have improved in recent years, titanium implant loosening remains a significant challenge facing orthopedic surgeons^15,16^. Osseointegration is a process through which implants are directly integrated into the bone tissue network structurally and functionally, forming a stably connected entity^17–20^.

Trichostatin A (TSA) was the first histone deacetylase inhibitor (HDACi) family drug to be used in the clinic as a cancer treatment^21,22^. Functionally, TSA can suppress class I and II histone deacetylase (HDAC) activity by binding zinc ions within these target enzymes^23^. In so doing, TSA can ultimately increase histone acetylation levels within treated cells, thus introducing heritable epigenetic modifications that alter cellular function^24^. As a pan-HDACi, TSA has been shown to enhance bone mesenchymal stem cells (BMSCs) differentiation into osteoblasts in rats via the inhibition of NF‐κB p65 binding to the DNA, in addition to promoting improved periodontal repair^25,26^. TSA has further been reported to augment the activity of osteoblasts, at least in part, by reducing intracellular levels of ROS^27^. TSA may thus represent a promising novel anabolic agent for treating diseases associated with bone loss. The potential of TSA to enhance the osseointegration of titanium rods remains an area for exploration. This study aimed to investigate whether TSA's potential in protecting osteoblasts from oxidative stress damage by activating the AKT/Nrf2 pathway. Moreover, these analyses demonstrated TSA's capability to enhance titanium rod integration in an ovariectomized (OVX) rat model of OP, marking a novel observation. These results suggest that TSA holds promise as a potential therapeutic agent for OP-related fractures, although further studies are imperative.

## Methods

### Reagents and instrumentation

Micro computed tomography (Micro-CT, ScancoMedical, Switzerland); Medical Slow DC Drill (Erbrigh-instrumente, Germany); Titanium rod implants (Huatrau, 1.5 × 10.0 mm, China); TSA (APExBIO, USA); Microcomputer-controlled electronic universal testing machine (Reger, China); Hematoxylin–eosin (HE) Stain Kit (Boster, China); Masson's Trichrome Stain Kit (Ribo, China); Immunohistochemistry kits (for Rabbit Primary Antibody) (Bioss ANTIBODIES, China); Digital pathology Slide Scanner (Motic Easy Scan, China); fetal bovine serum (Gibco, USA); penicillin–streptomycin solution (Beyotime, China); α-MEM medium (Gibco, USA); β-glycerophosphate (Solarbio, China); ascorbic acid (Solarbio, China); dexamethasone (Solarbio, China); Alkaline phosphatase (ALP) Assay Kit (Beyotime, China); inverted fluorescence microscope (Nikon; Japan); ALP activity Assay Kit (Solarbio, China); Bicinchonininc acid (BCA) protein Assay Kit (Beyotime, China); Alizarin red S (ARS) Solution (Solarbio, China); cetylpyridinium chloride (Solarbio, China); Microplate Reader (Bio Tek Instruments, USA); carbonyl cyanide 3-chlorophenylhydrazone (CCCP) (Solarbio, China); LY294002 (MCE, USA); Cell Counting Kit-8 (CCK8) (Beyotime, China); Enhanced Mitochondrial Membrane Potential Assay Kit with JC-1 (Beyotime, China); Antifade Mounting Medium with DAPI (Beyotime, China); Malondialdehyde (MDA) Assay Kit (Nanjing Jiancheng Bioengineering Institute, China); Superoxide Dismutase (SOD) Activity Assay Kit (Nanjing Jiancheng Bioengineering Institute, China); ROS Assay Kit (Beyotime, China); flow cytometry (Beckman Coulter, USA); Annexin V-FITC Apoptosis Detection Kit (Beyotime, China); Nuclear and Cytoplasmic Protein Extraction Kit (Beyotime, China); nanoscale titanium powder (99.8%, 60 nm) (Macklin, China); titanium sheet (0.1 mm thick, 34 mm in diameter) (baoti, China); electron microscope (hitachi, Japan).

### Cell culture and treatment

The MC3T3-E1 Subclone 14 mouse calvarial preosteocyte cell line, sourced from CELLCOOK, Guangzhou, China, was used in this study. Cells were cultured in base medium (10% fetal bovine serum, 1% penicillin–streptomycin, α-MEM). An oxidative stress model was established by treating cells with carbonyl cyanide 3-chlorophenylhydrazone (CCCP; 10 μM) for 10 min. In total, 6 treatment groups were established: NC group (untreated normal control cells); CCCP group (10 μM CCCP treatment for 10 min); TSA group (8 μM TSA treatment for 24 h); CCCP + TSA group (10 μM CCCP treatment for 10 min, 8 μM TSA treatment for 24 h); TSA + LY294002 group (8 μM TSA treatment for 24 h, 50 μM LY294002 treatment for 24 h); CCCP + TSA + LY294002 group (10 μM CCCP treatment for 10 min, 8 μM TSA treatment for 24 h, 50 μM LY294002 treatment for 24 h).

### CCK-8 assay

CCK-8 was used for rapid and sensitive detection of cell proliferation and cytotoxicity. MC3T3-E1 cells in 96-well plates were treated with varying TSA concentrations (0, 1, 3, 5, 8, 10, 20, 50, 100 μM) over time intervals (6, 12, 24, 48, and 72 h), followed by the addition of 10 μL CCK-8 reagent per well. After an additional 2 h incubation, absorbance was analyzed at 450 nm using a microplate reader, with absorbance values being used to establish cell survival rates.

### Analysis of oxidative stress-related biomarkers

Superoxide dismutase (SOD) is vital for antioxidant defense, and malondialdehyde (MDA), a lipid peroxidation byproduct, is a marker for oxidative stress levels in cells. Detecting SOD and MDA concentrations can reflect the degree of oxidative stress from different perspectives. MC3T3-E1 cells were cultured in 24-well plates and treated as above, following adherence to the plate. Following a 6-day incubation period, cells were harvested, and MDA and SOD analyses were performed to gauge oxidative stress levels using MDA Assay and SOD Assay kits based on provided directions. A BCA protein assay kit was used to measure protein levels in each sample.

In another experiment, post a 6-day treatment regimen as previously described, a ROS Assay Kit utilizing 2′,7′-dichlorodihydro-fluorescein diacetate (DCFH-DA) was employed to evaluate ROS levels according to the manufacturer's instructions. ROS converts DCFH-DA that enters cells into green fluorescent substances. Therefore, when observing the cells from a fluorescence microscope, the stronger the green fluorescence, the higher the ROS content.

In addition, flow cytometry was utilized to quantify mean fluorescence intensity values as a measure of ROS production in these cells.

### Analyses of MMP

The JC-1 probe, emitting red or green fluorescence to indicate low or high MMP levels, was employed to detect MMP. MC3T3-E1 cells were cultured in 24-well plates and treated as above, following adherence to the plate. After treatment, cells were rinsed thrice with PBS, fixed for 10 min using a neutral tissue fixator, and stained with JC-1 based on the provided kit instructions. Nuclei were subsequently stained with 100 μl of a prepared DAPI solution, and cells were imaged with a fluorescence microscope, followed by analysis using ImageJ software (version 1.52v, NIH, USA).

### Apoptosis analyses

Following the abovementioned treatment, cells were stained with Annexin V-FITC and PI using an Apoptosis Detection Kit and analyzed through flow cytometry. Early and late apoptotic cells were defined as FITC+/PI− and FITC+/PI+ cells, respectively, and the sum of these two cell types was determined to establish the overall apoptosis rate.

### Western blot

Cells were washed with chilled PBS and lysed using RIPA buffer for 15 min following the abovementioned treatment. Lysates were then transferred to 1.5 mL Eppendorf tubes and centrifuged for 30 min at 15,000 rpm at 4 °C. Lysates were combined with 5× protein loading buffer at a 4:1 ratio and boiled for 12 min to facilitate protein denaturation. Samples were then separated via SDS-PAGE and transferred to PVDF membranes, and these blots were blocked using 5% BSA for 2 h. Blots were then probed overnight using antibodies specific for Nrf2, AKT, HO-1, NQO1, OPN, Runx2, BMP2, OCN, Caspase3, Bcl2, Bax, cleaved caspase-3, and β-actin, at 4 °C with constant shaking. The following day, blots were rinsed with TBST and probed for 2 h with appropriate secondary antibodies. After an additional wash with TBST, protein bands were detected with a luminescent ECL solution, and densitometric analyses were conducted using ImageJ. Blots were sectioned before antibody hybridization, precluding the acquisition of full-length blot images. However, images of all blots, inclusive of visible membrane edges and replicates, are available in the Supplementary material.

### Nuclear and cytoplasmic protein fractionation assays

For nuclear and cytoplasmic protein fractionation assays, three MC3T3-E1 cell groups were delineated: CCCP group, CCCP + TSA group, and CCCP + TSA + LY294002 group. Post-treatment, a Nuclear and Cytoplasmic Protein Extraction Kit facilitated the isolation of cytosolic and nuclear proteins, which were then subjected to Western blot analysis for total, nuclear, and cytosolic Nrf2.

### Analyses of MC3T3-E1 cell nanoscale titanium powder absorption, fusion, and osteogenic ability

MC3T3-E1 cells, post adherence to 24-well plates, underwent various treatments with nanoscale titanium powder. The groups included: CCCP group (10 μM CCCP for 10 min); CCCP + TI group (10 μM CCCP for 10 min, 0.1 mg/ml nanoscale titanium powder); CCCP + TI + TSA group (10 μM CCCP for 10 min, 0.1 mg/ml nanoscale titanium powder, 8 μM TSA for 24 h); CCCP + TI + TSA + LY294002 group (10 μM CCCP for 10 min, 0.1 mg/ml nanoscale titanium powder, 8 μM TSA for 24 h, 50 μM LY294002 for 24 h).

After treatment, Western blotting was performed. Following 7 days post-osteogenesis induction, ALP staining was conducted to assess differentiation.

### Analyses of MC3T3-E1 cell attachment to a titanium sheet

Sterile titanium sheets (0.1 mm thick, 24 mm diameter) were placed in the wells of a 6-well plate, serving as a substrate for seeding MC3T3-E1 cells under various treatment conditions. Treatment conditions for the cells included: TS + CCCP group (10 μM CCCP for 10 min); TS + CCCP + TSA group (10 μM CCCP for 10 min, 8 μM TSA for 24 h); TS + CCCP + TSA + LY294002 group (10 μM CCCP for 10 min, 8 μM TSA for 24 h, 50 μM LY294002 for 24 h). After treatment, electron microscopy was used to assess MC3T3-E1 cell adhesion to the titanium surface.

### Experimental animals

The Ethical Committee of Yijishan Hospital approved all studies. Female Sprague–Dawley rats, aged 12 weeks and weighing 220–240 g, were housed in Yijishan Hospital's Central Laboratory. They were maintained under standard environmental conditions (22 ± 2 °C, 50% humidity, and a 12-h light/dark cycle) with unrestricted access to food and water. The rats were euthanized under isoflurane anesthesia.

### Ethics statement

All animal experiments were approved by the Scientific Research and New Technology Ethics Committee of Yijishan Hospital, Wannan Medical College ((2021) Ethical Review Approval No. 071) and conducted in accordance with the relevant guidelines. The study was carried out in compliance with the ARRIVE guidelines.

### Surgery and treatment

Sixty rats were divided into two groups: a sham surgery group (n = 15) and a bilateral OVX group (n = 45). The surgical procedure involved making a 1.5 cm skin incision 2 cm below the costal arch and 1.5 cm from the spine. The subcutaneous tissue, muscle, and fascia were bluntly separated, exposing the white fat tissue. Upon manipulating this fat tissue, the mulberry-like ovary, approximately 0.5 cm in diameter, became visible. The junction between the oviduct and the ovary was resected with suitable suturing, followed by the removal of the ovary. This process was repeated for both ovaries, after which the incisions were closed using sutures. Postoperatively, rats were administered intramuscular penicillin (200,000 IU/mL, 1 mL/kg) for three days. Identical operative procedures were performed on the sham control group, except that only para-ovarian fatty tissue was removed. At 3 months post-surgery, 5 rats from each group underwent random selection and euthanasia to confirm successful OP model establishment. Subsequently, the remaining 50 rats were utilized for distal femur bone defect modeling. Briefly, the skin on the medial side of the knee joint was incised, followed by the femur medial condyle exposure following the blunt separation of the subcutaneous, muscle, and fascial tissue. A 1 mm circular bone defect was created using a medical DC drill, penetrating the femur lateral condyle. Next, 20 total rats were randomly selected to undergo the implantation of a titanium rod (diameter: 1.5 mm) at the site of this bone defect. The 5 groups of rats after these surgical procedures are: CON group (n = 10, sham surgery, bone defect surgery, and normal saline treatment); BD group (n = 10, OVX, bone defect surgery, and normal saline treatment); TSA group (n = 10, OVX, bone defect surgery, and 500 μg/kg/day TSA treatment); TI group (n = 10, OVX, bone defect surgery, titanium implants, and normal saline treatment); TSA + TI group (n = 10, OVX, bone defect surgery, titanium implants, and 500 μg/kg/day TSA treatment). Daily intraperitoneal injections of drugs or equivalent volumes of normal saline were administered for 8 weeks. Rats underwent daily weighing, and injected volumes were adjusted based on each rat's weight.

### Micro-CT analyses

Micro-CT analysis of the rats' left femur focused on the area around the titanium rod implantation site. The region of interest (ROI) was defined as a 2 mm diameter cylinder. After 3D reconstruction of the ROI, software analysis measured parameters such as BMD, bone volume fraction (BV/TV), trabecular number (Tb.N), trabecular thickness (Tb.Th), and trabecular separation (Tb.Sp).

### Axial pull-out force analyses

Post-euthanasia, femurs from the TI and TSA + TI groups were collected for analysis. Surrounding soft tissue was removed to reveal the heads of the implanted screws. Subsequently, a paste consisting of 20 g of dental powder and 4 mL of PBS was prepared, and a retention frame was used to stabilize the femur. Following the dental paste's 40-min solidification, the retention frame's clamping blade secured the head of the titanium rod. The fixing mechanism was connected to an electronic universal testing machine, with its long axis being aligned to the titanium rod. It was then pulled at 2.0 mm/min, and GraphPad Prism was used to analyze the resultant data.

### Histological and immunohistochemical analysis

The right femur of each rat was decalcified, sectioned, and embedded in paraffin, after which H&E, Masson's trichrome staining, and immunohistochemical analysis were performed based on the instructions provided with appropriate kits. Masson's trichrome staining resulted in muscle fibers staining red, whereas blue staining highlighted collagen fibers and new bone tissue. Immunohistochemistry was employed to detect the expression levels of pathway-related protein AKT and osteogenesis-associated protein OCN in the femur. AKT and OCN served as primary antibodies, while Goat-anti-Rabbit-IgG was applied as the secondary antibody. Antigen-expressing positive cells were stained brown. Photographs were obtained under a light microscope. The average optical density (AOD) of AKT and OCN was assessed using ImageJ software. A digital pathological section scanner was used to scan the resultant sections.

Histological and immunohistochemical analyses were conducted after two months of drug treatment. After fixation, decalcification, and paraffin embedding, the femur was incised into 5 μm slices and stained with HE and Masson's trichrome stains for morphologic assessment. New bone formation after bone injuries was examined using a light microscope (Nikon Eclipse Ti-U; USA). After Masson's trichrome staining, new bone tissue and collagen fibers were stained blue, and mature bone and muscle fibers were stained red. The new bone formation in the defect area was quantified using ImageJ software.

Osteocalcin (OCN) and type 1 collagen (COL1) expression levels in the femur were detected using immunohistochemistry. Briefly, slices were deparaffinized with xylene, immersed in 3% H2O2 to remove endogenous catalase, blocked against non-specific antibodies, and then incubated with specific primary antibodies (OCN, 1:200, DF12303, Affinity; COL1, 1:100, AF7001, Affinity) overnight at 4 °C. The next day, the slices were incubated with the secondary antibody, subjected to DAB staining, then counterstained with hematoxylin, dehydrated, and mounted. Antigen-expressing positive cells were stained brown. Photographs were obtained under a light microscope. The average optical density of OCN and COL1 were measured by ImageJ software.

### Evaluation of BMSCs' osteogenic ability

Post-euthanasia, rat bodies from each treatment group were sterilized with 75% ethanol for BMSCs extraction. The skin and muscle were swiftly removed from the animals' hind limbs to expose the femurs. These were then placed in PBS within centrifuge tubes. The tubes had been sterilized with a 30-min ultraviolet light treatment. Femurs were then transferred to an ultraclean table, after which they were washed thrice with PBS supplemented with 1% penicillin–streptomycin. The epiphyses at both ends of the femur were removed, exposing the inner bone marrow cavity, which was then repeatedly flushed with base medium using a 5 mL syringe. The resultant cell-containing medium was transferred to 100 mm cell culture dishes. After 48 h, non-adherent cells were removed. Cells were then cultured to 80% confluence for subsequent use.

The osteogenic induction medium, composed of 50 mL of the base medium supplemented with 10 mM β-glycerophosphate, 50 μM ascorbic acid, and 100 nM dexamethasone, was used for culturing BMSCs. Briefly, BMSCs from the third passage were seeded in 24-well plates, and the prepared osteogenic medium was added to each well. Medium was replaced every 3 days.

At 1 week after osteogenic induction, ALP staining was performed. Briefly, the osteogenic medium was removed from all wells, and cells were rinsed thrice with PBS, followed by fixation for 15 min using paraformaldehyde. After three additional washes with PBS, the BCIP/NBT staining solution was prepared based on the instructions provided with an ALP assay kit and added to each well. Cells were incubated in the dark at room temperature for 30 min, after which the staining solution was removed, and cells were washed thrice with PBS prior to imaging with an inverted microscope. ALP Activity Detection and BCA Protein Assay kits were then used based on the provided direction for quantifying ALP activity levels.

At 3 weeks after the start of osteogenic induction, ARS staining was initiated. Briefly, the osteogenic medium was removed from all wells, and cells were rinsed thrice with PBS, followed by fixation for 15 min using paraformaldehyde. After three additional washes with PBS, prepared ARS solution was added in each well, and plates were incubated at room temperature for 30 min, after which the staining solution was removed, and cells were washed thrice with PBS prior to imaging with an inverted microscope. Lastly, calcium nodules were dissolved using 10% cetylpyridinium chloride, and absorbance at 570 nm was analyzed via a microplate reader.

### Statistical analyses

Data expressed as mean ± standard deviation (SD) were analyzed with GraphPad Prism (USA) using independent samples t-tests and one-way ANOVAs; P < 0.05 was set as the significance threshold.

## Results

### CCK-8 analyses of MC3T3-E1 cells

**Figure 1:**
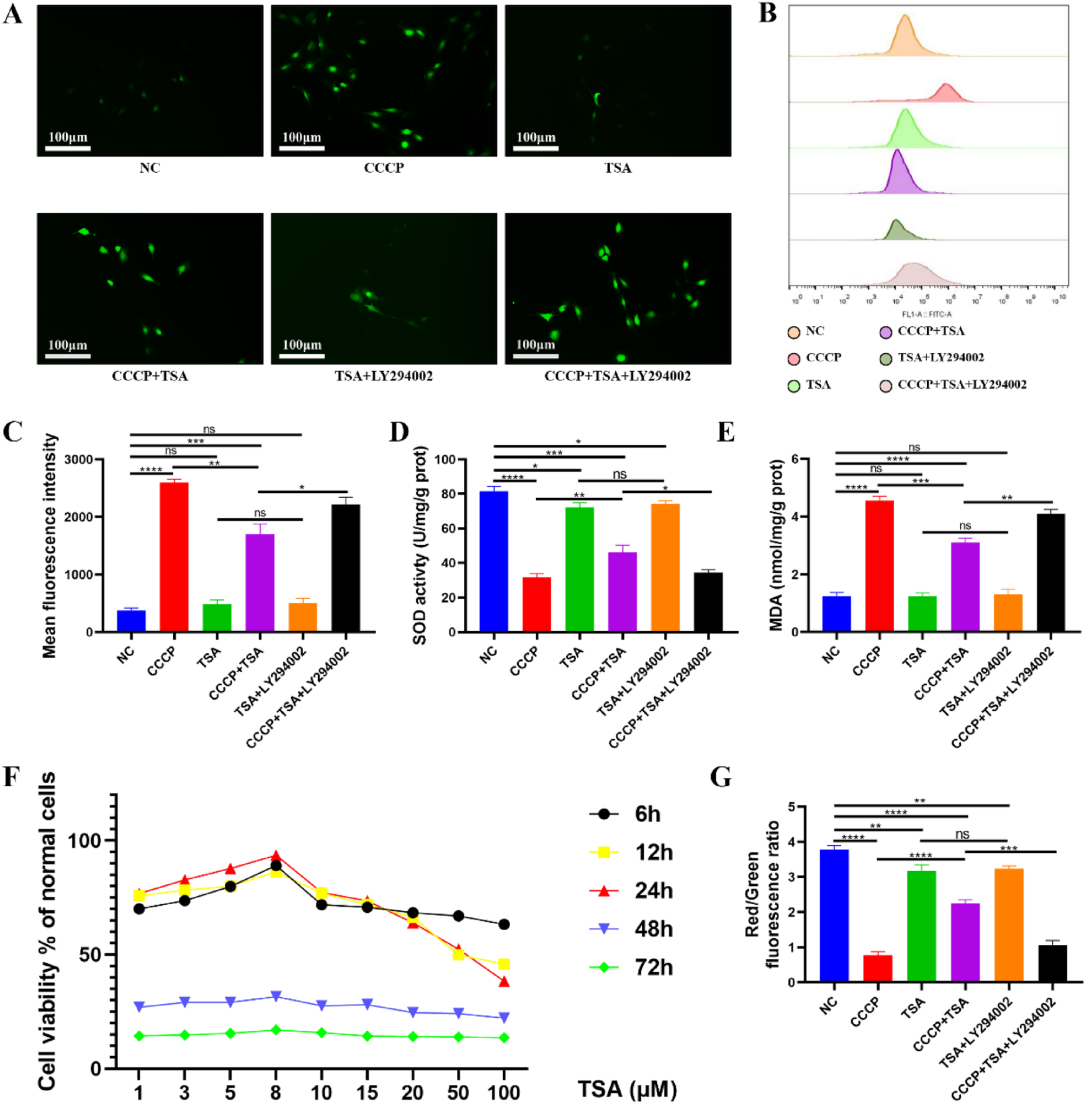
Effects of different treatments on oxidative stress indexes of MC3T3-E1 cells. (**A**) ROS images were detected by fluorescence microscope, scale bar = 100 μm; (**B**) ROS was detected by flow cytometry; (**C**) The average fluorescence intensity of ROS was determined by flow cytometry; (**D**) Quantitative analysis of SOD activity; (**E**) Quantitative analysis of MDA content; (**F**) CCK8 cell activity detection; (**G**) JC-1 fluorescence quantitative analysis (*P < 0.05, **P < 0.01, ***P < 0.001, ****P < 0.0001).

MC3T3-E1 cell viability initially increased with increasing TSA concentrations and then decreased, with a peak at 8 μM (Fig. 1F). At different time points, the viability of MC3T3-E1 cells reached its highest at 24 h, recording a peak value of 93.5%.

### Analyses of oxidative stress indices in MC3T3-E1 cells

Compared to NC cells, CCCP-treated cells showed higher ROS fluorescence (Fig. 1A–C), lower SOD activity (Fig. 1D), and increased MDA content (Fig. 1E). The oxidative stress indices showed no significant difference between the TSA and NC treatment groups. However, significant reductions in ROS fluorescence intensity were observed in the CCCP + TSA group as compared to the CCCP group, with corresponding reductions in MDA content and higher levels of SOD activity. While the TSA and TSA + LY294002 groups exhibited no variation in these indices, the CCCP + TSA + LY294002 group experienced a significant rise in ROS fluorescence intensity, reduced SOD activity, and increased MDA content compared to the CCCP + TSA group.

### Analyses of MC3T3-E1 cell MMP

**Figure 2:**
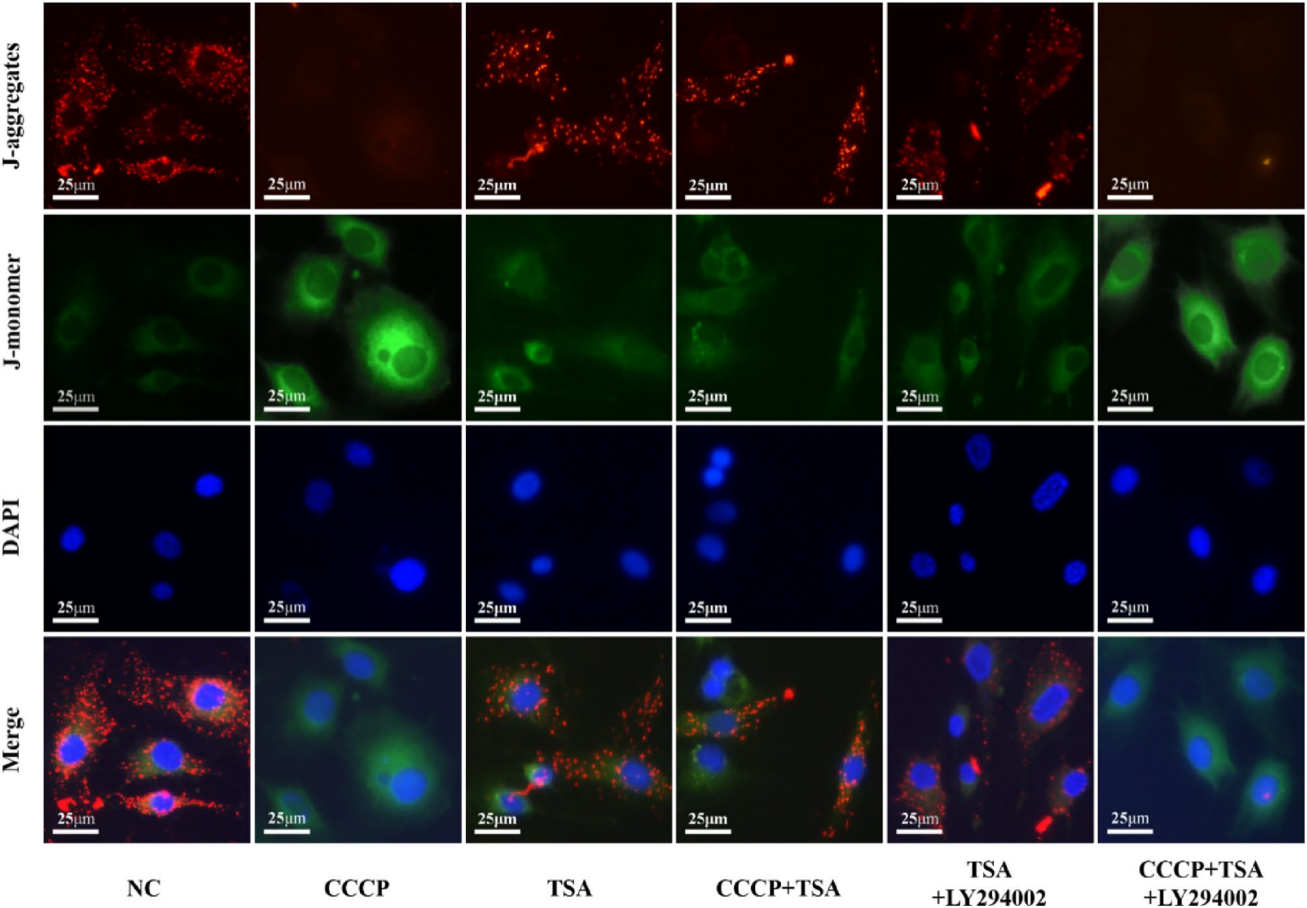
Effects of different treatments on MMP of MC3T3-E1 cells. (JC-1 staining image, scale bar = 25 μm).

Untreated cells in the NC group exhibited strong red JC-1 fluorescence and a high red/green fluorescence ratio (Figs. 1G, 2), whereas CCCP treatment was associated with higher levels of green fluorescence and a reduction in the red/green ratio. The TSA group displayed a noticeable decrease in MMP compared to the NC group. Increased red fluorescence and a higher red/green ratio were evident in cells treated with CCCP + TSA relative to those treated with CCCP alone. No significant differences were evident between the TSA and TSA + LY294002 groups. The CCCP + TSA + LY294002 group showed reduced red fluorescence and a lower red/green fluorescence ratio than the CCCP + TSA group.

### The impact of treatments on MC3T3-E1 cell apoptosis

**Figure 3:**
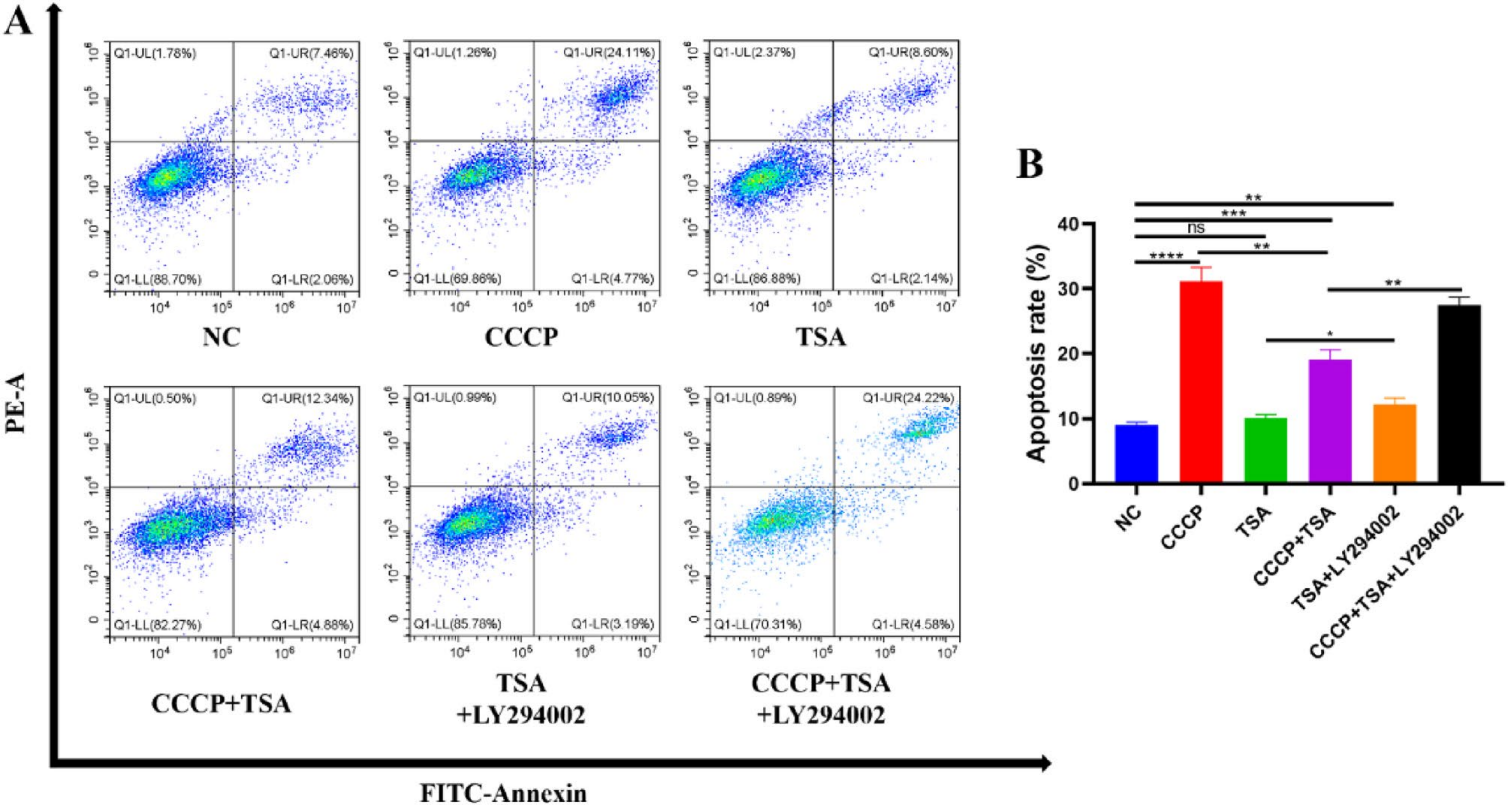
Effects of different treatments on apoptosis rate of MC3T3-E1 cells. (**A**) Results of cell apoptosis rate detected by flow cytometry; (**B**) analysis of the results of apoptosis rate (*P < 0.05, **P < 0.01, ***P < 0.001, ****P < 0.0001).

Apoptosis rates were higher in the CCCP group compared to the NC group, while the NC, TSA, and TSA + LY294002 groups showed no significant differences (Fig. 3). However, a significant reduction in apoptosis was evident in the CCCP + TSA group relative to the CCCP group. In contrast, the rate of apoptotic death for cells treated with CCCP + TSA + LY294002 increased relative to that for cells treated with CCCP + TSA.

### The impact of TSA on MC3T3-E1 cell protein expression

**Figure 4:**
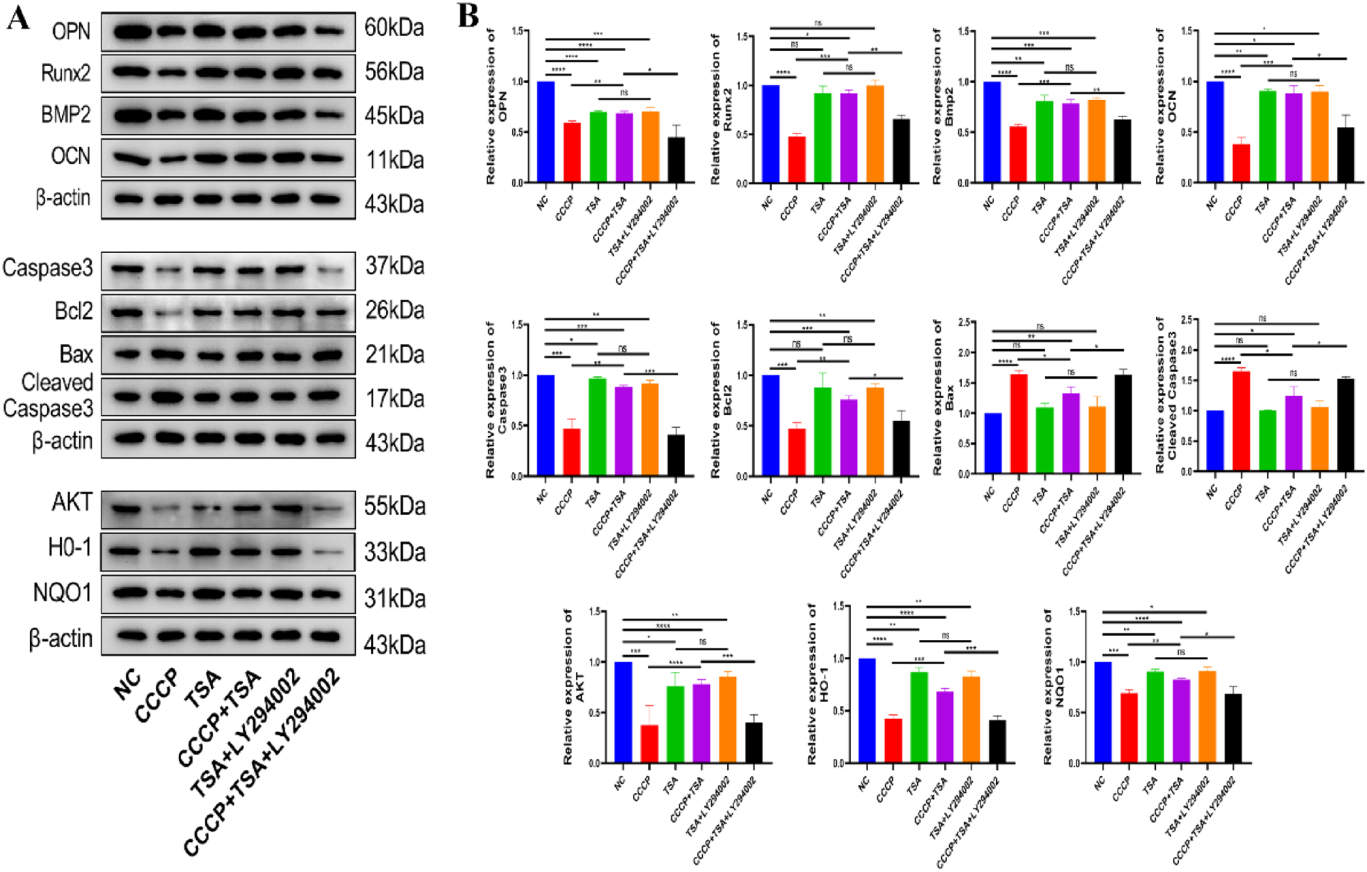
Effects of different treatments on protein expression of MC3T3-E1 cells. (**A**) Representative bands of osteogenesis-related proteins (OPN, Runx2, BMP2, and OPN), apoptosis-related proteins (Caspase3, Bcl2, Bax, and Cleaved Caspase3), and some pathway-related proteins (AKT, HO-1, and NQO1); (**B**) Relative expression levels of related proteins in each group compared with NC group. Original blots are presented in Supplementary Panel A (*P < 0.05, **P < 0.01, ***P < 0.001, ****P < 0.0001).

CCCP treatment resulted in significant decreases in levels of the osteogenesis-associated proteins Runx2, BMP2, and OPN, together with increased Bax and cleaved caspase-3 levels, and reduced levels of HO-1, NQO1, AKT, total caspase-3 and Bcl-2, compared to the NC group (Fig. 4). No significant differences in protein levels were evident when comparing the TSA and NC groups. Osteogenesis-associated protein levels rose in the CCCP + TSA group as compared to the CCCP group, whereas Bax and cleaved caspase-3 levels decreased, and levels of total caspase-3, Bcl-2, AKT, HO-1, and NQO1 increased. These protein expression changes were reversed with the concurrent administration of LY294002, CCCP, and TSA.

### The impact of TSA treatment on Nrf2 subcellular localization

**Figure 5:**
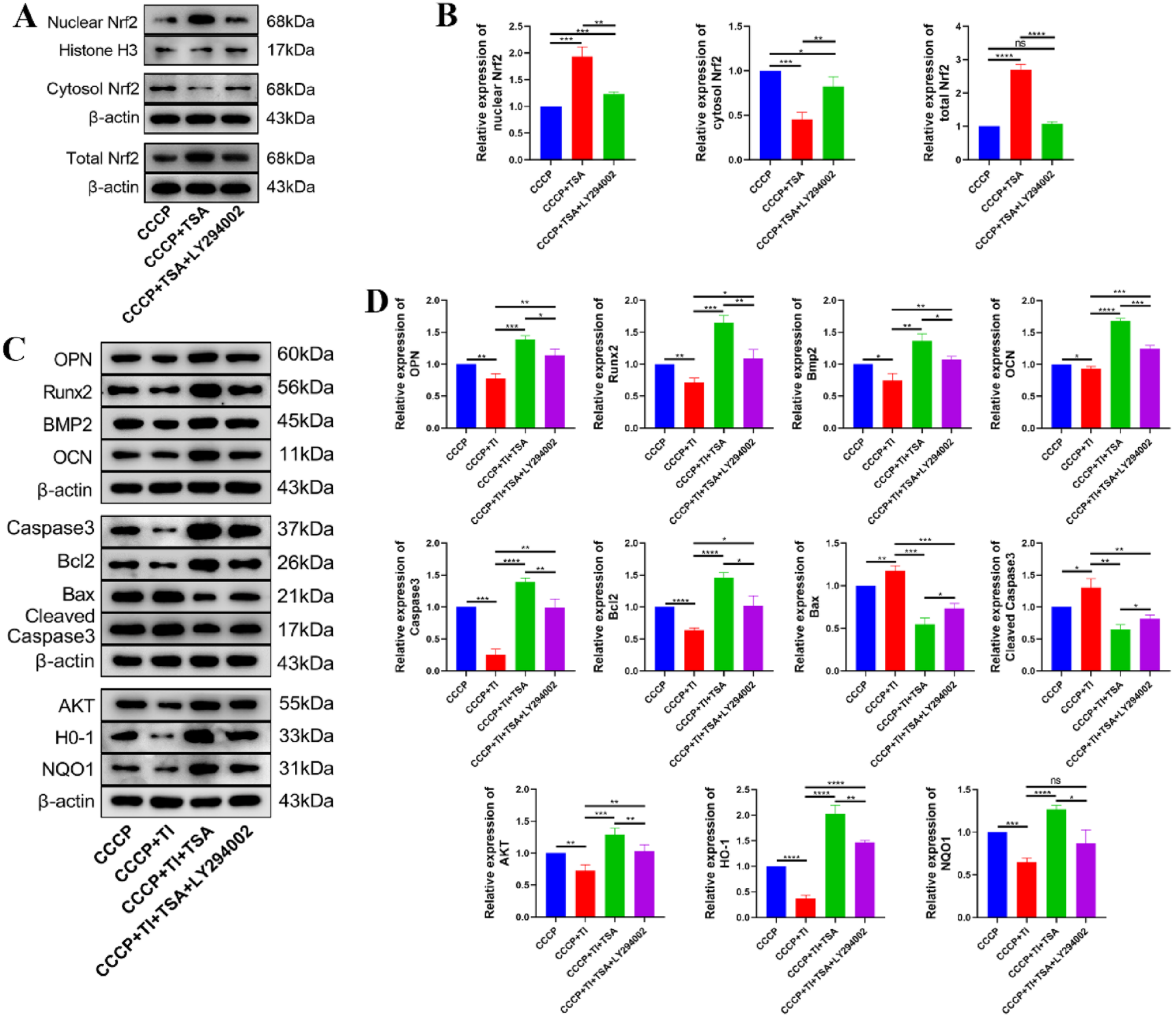
The effects of TSA on Nrf2 and the effects of different treatments on protein expression of MC3T3-E1 cells after adding titanium powder were detected by nuclear cytoplasmic separation. (**A**) Representative expression bands of Nrf2 in nucleus, cytoplasm and total Nrf2; (**B**) Histogram of relative expression levels of Nrf2 in nucleus, cytoplasm and total Nrf2; (**C**) Representative bands of osteogenesis-related proteins (OPN, Runx2, BMP2, and OPN), apoptosis-related proteins (Caspase3, Bcl2, Bax, and Cleaved Caspase3), and some pathway-related proteins (AKT, HO-1, and NQO1) in MC3T3-E1 cells after adding titanium powder; (**D**) Relative expression levels of related proteins in each group compared with NC group. Original blots are presented in Supplementary Panel B and C (*P < 0.05, **P < 0.01, ***P < 0.001, ****P < 0.0001).

Compared to the CCCP group, CCCP + TSA treatment increased nuclear and overall Nrf2 expression while reducing cytosolic Nrf2 (Fig. 5A,B). Relative to CCCP + TSA treatment, cells treated with CCCP + TSA + LY294002 exhibited lower levels of nuclear and overall Nrf2 expression together with higher levels of cytosolic Nrf2.

### Analyses of altered gene expression in MC3T3-E1 cells following nanoscale titanium powder treatment

In the CCCP + TI group, there were slight reductions in osteogenesis-related proteins, including OPN, BMP2, and Runx2, and increases in apoptosis markers, including cleaved caspase-3 and Bax, lower levels of AKT, HO-1, and NQO1 compared to the CCCP group (Fig. 5C,D). Furthermore, these cells demonstrated reduced levels of overall caspase-3 and Bcl-2. As compared to cells in the CCCP + TI group, those in the CCCP + TI + TSA group exhibited higher levels of osteogenesis-associated protein expression, reduced cleaved caspase-3 and Bax expression, and higher levels of caspase-3, Bcl-2, AKT, HO-1, and NQO1. Subsequent LY294002 treatment reversed these changes in Bax, cleaved caspase-3, total caspase-3, Bcl-2, HO-1, NQO1, and AKT.

### Analyses of MC3T3-E1 cell fusion, absorption, and osteogenic ability when exposed to nanoscale titanium powder

**Figure 6:**
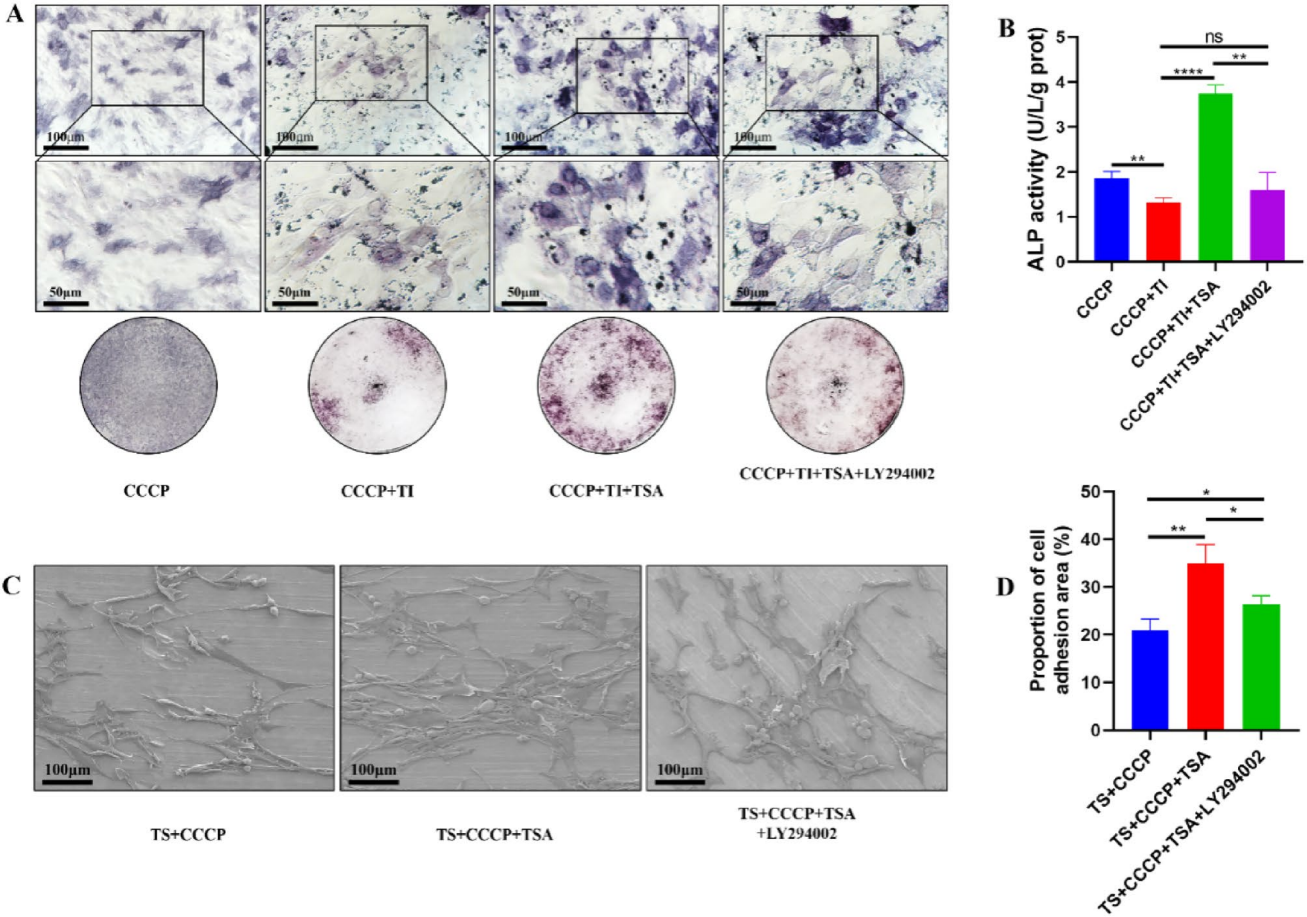
Effects of different treatments on osteogenic ability of MC3T3-E1 cells after adding titanium powder and results of MC3T3-E1 cells attached on a titanium sheet. (**A**) Results of ALP staining, scale bar = 100 μm, scale bar = 50 μm; (**B**) ALP activity diagram; (**C**) Images of electron microscopy; (**D**) Proportion of MC3T3-E1 cell adhesion area (*P < 0.05, **P < 0.01, ***P < 0.001, ****P < 0.0001).

Compared to the CCCP group, MC3T3-E1 cells in the CCCP + TI group exhibited reduced ALP activity with less blue-purple staining. In the CCCP + TI + TSA group, an increase in both titanium particle incorporation and ALP activity was observed compared to the CCCP + TI group, as evidenced by darker staining (Fig. 6A,B). However, this effect was mitigated when LY294002 was added to the treatment, leading to fewer titanium particles, lighter staining, and lower ALP activity. Notably, no significant differences were detected between the CCCP + TI and CCCP + TI + TSA + LY294002 groups in these parameters.

### MC3T3-E1 cell attachment to a titanium sheet

In the TS + CCCP groups, MC3T3-E1 cells were elongated, misshapen, and atrophied, demonstrating decreased adherence (Fig. 6C,D). In contrast, cells in the TS + CCCP + TSA group maintained a normal morphology, exhibiting enhanced attachment to titanium sheets. However, LY294002 reversed the beneficial effects of TSA in this assay setting, with TS + CCCP + TSA + LY294002 cells appearing deformed, atrophic, and less adherent.

### Verification of rat OP model establishment

**Figure 7:**
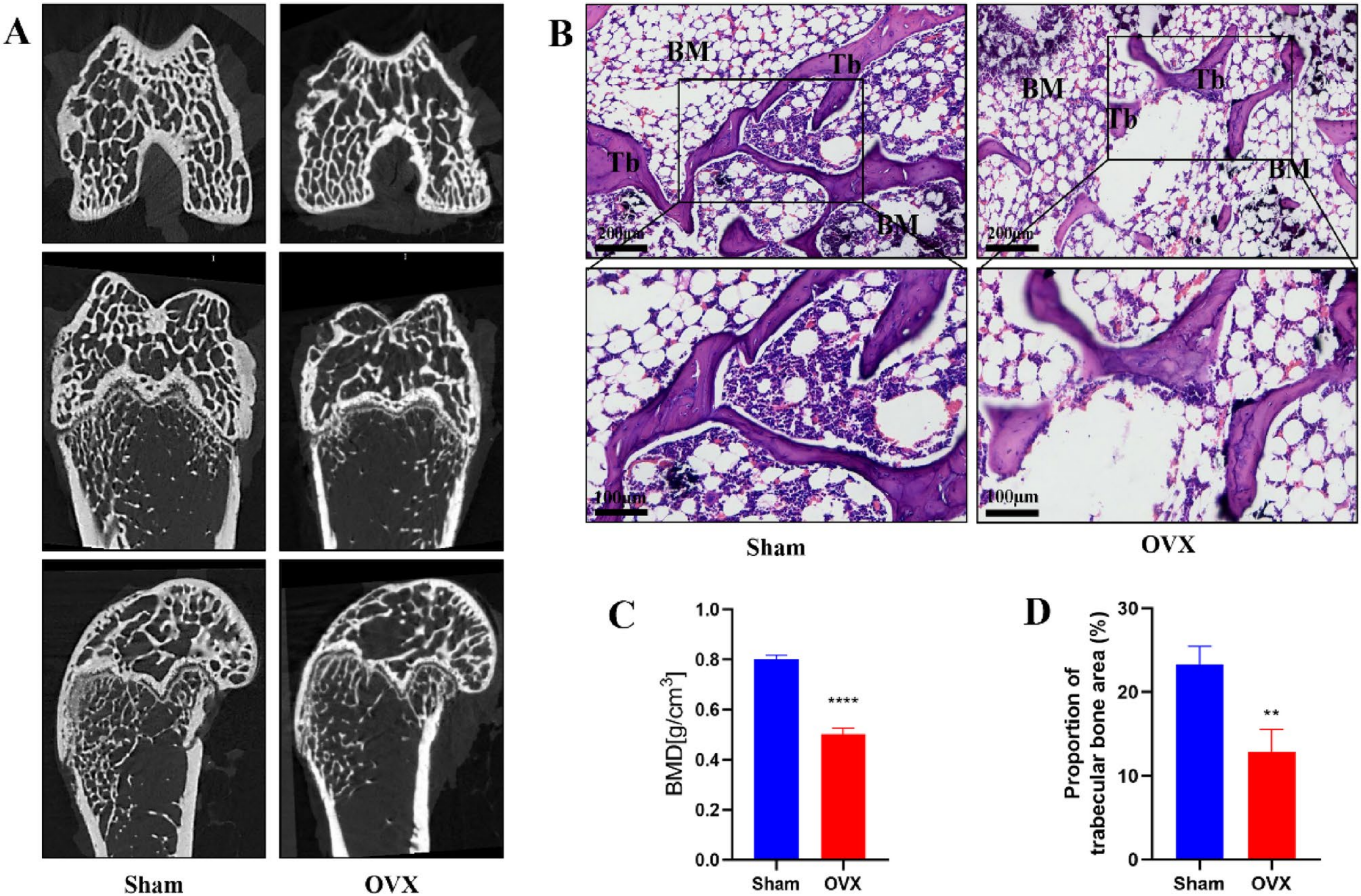
Verification of rat OP model establishment. (**A**) Micro-CT images at femur metaphysis in Sham group and OVX group; (**B**) HE staining images at femur metaphysis in Sham group and OVX group, Scale bar = 200 μm; (**C**) Quantitative analysis of BMD at femur metaphysis; (**D**) Proportion of trabecular bone area in ROI. Tb: Trabecular bone. BM: bone marrow (**P < 0.01, ****P < 0.0001).

Micro-CT scans confirmed significant reductions in trabecular bone volume in the femoral epiphysis of OVX rats, accompanied by increased bone atrophy and fractures (Fig. 7A). Quantitative analyses revealed a significant decrease in BMD in the OVX group (P < 0.05) (Fig. 7C), while H&E staining also confirmed that these OVX model rats exhibited the abnormal thinning of the trabecular bone structure together with bone atrophy and fracture in some instances (Fig. 7B,D). Together, these results confirmed the successful establishment of a rat model of OP.

### Micro-CT scanning, reconstruction, and analyses

**Figure 8:**
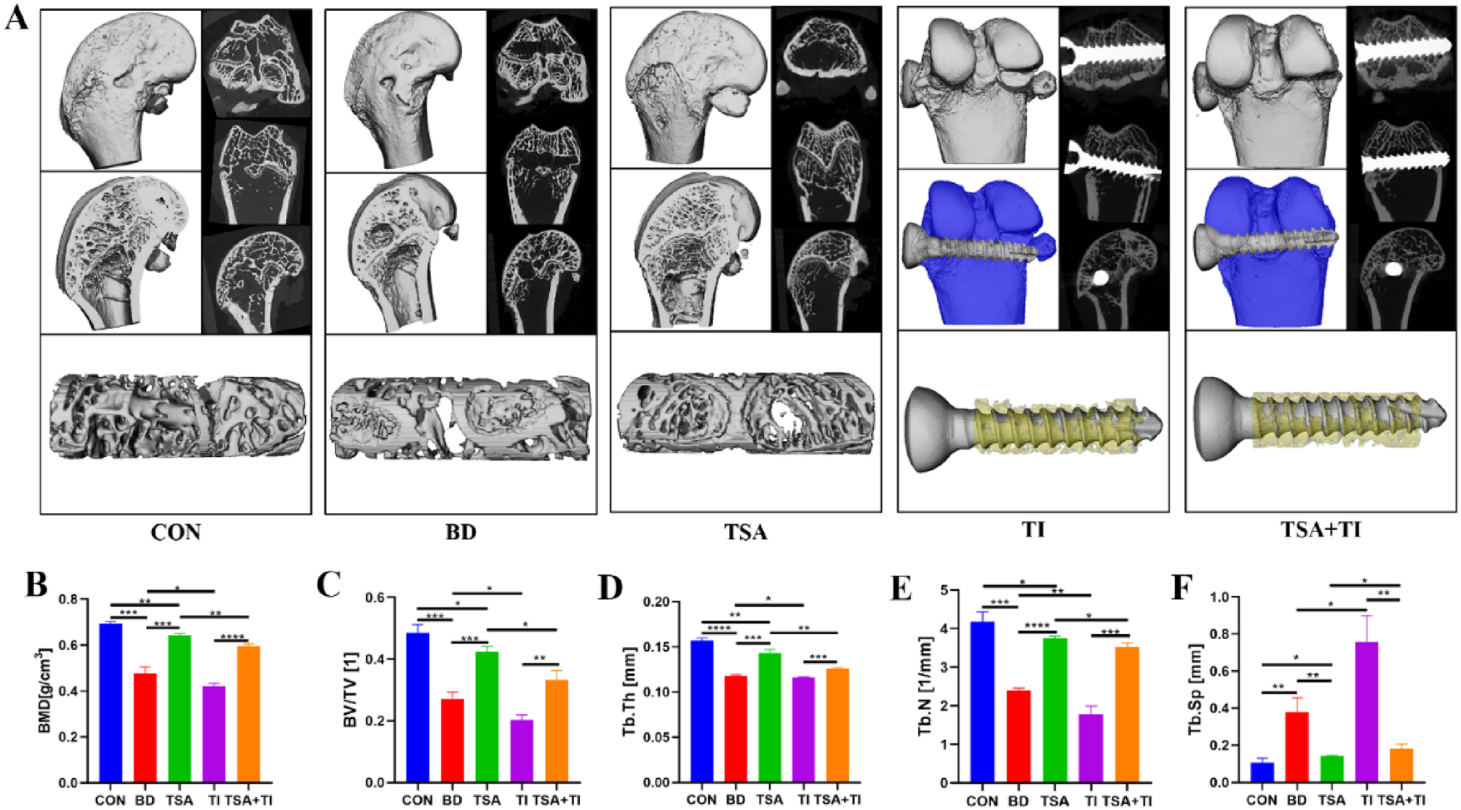
Results of micro-CT examination. (**A**) Three-dimensional reconstruction images of Titanium rods and surrounding tissues at femur metaphysis; (**B**) Quantitative analysis of ROI at femur Metaphysis (*P < 0.05, **P < 0.01, ***P < 0.001, ****P < 0.0001).

The BD group exhibited reduced trabecular bone content, heightened bone atrophy, and more fractures compared to the CON group (Fig. 8A). This deterioration corresponded with marked reductions in BMD, BV/TV, Tb.N, and Tb.Th, and a rise in Tb.Sp (P < 0.05) (Fig. 8B–F). Relative to the BD group, improved trabecular bone quality was evident in the TSA treatment group, along with increased BMD, BV/TV, Tb.N, and Tb.Th and reductions in Tb.Sp (P < 0.05) (Fig. 8B–F), although the trabecular bone quality was still lower than in the CON group. Following titanium rod implantation, significant reductions in metaphyseal trabecular numbers were evident in the TI group (Fig. 8A). Relative to the TI group, TSA treatment led to increases in BMD, BV/TV, Tb.N, and Tb.Th, as well as decreases in Tb.Sp (P < 0.05) (Fig. 8B–F), consistent with the increased transformation of rod-like bone trabeculae into plate-like structures indicative of enhanced new trabecular bone formation.

### Axial pull-out force analyses

**Figure 9:**
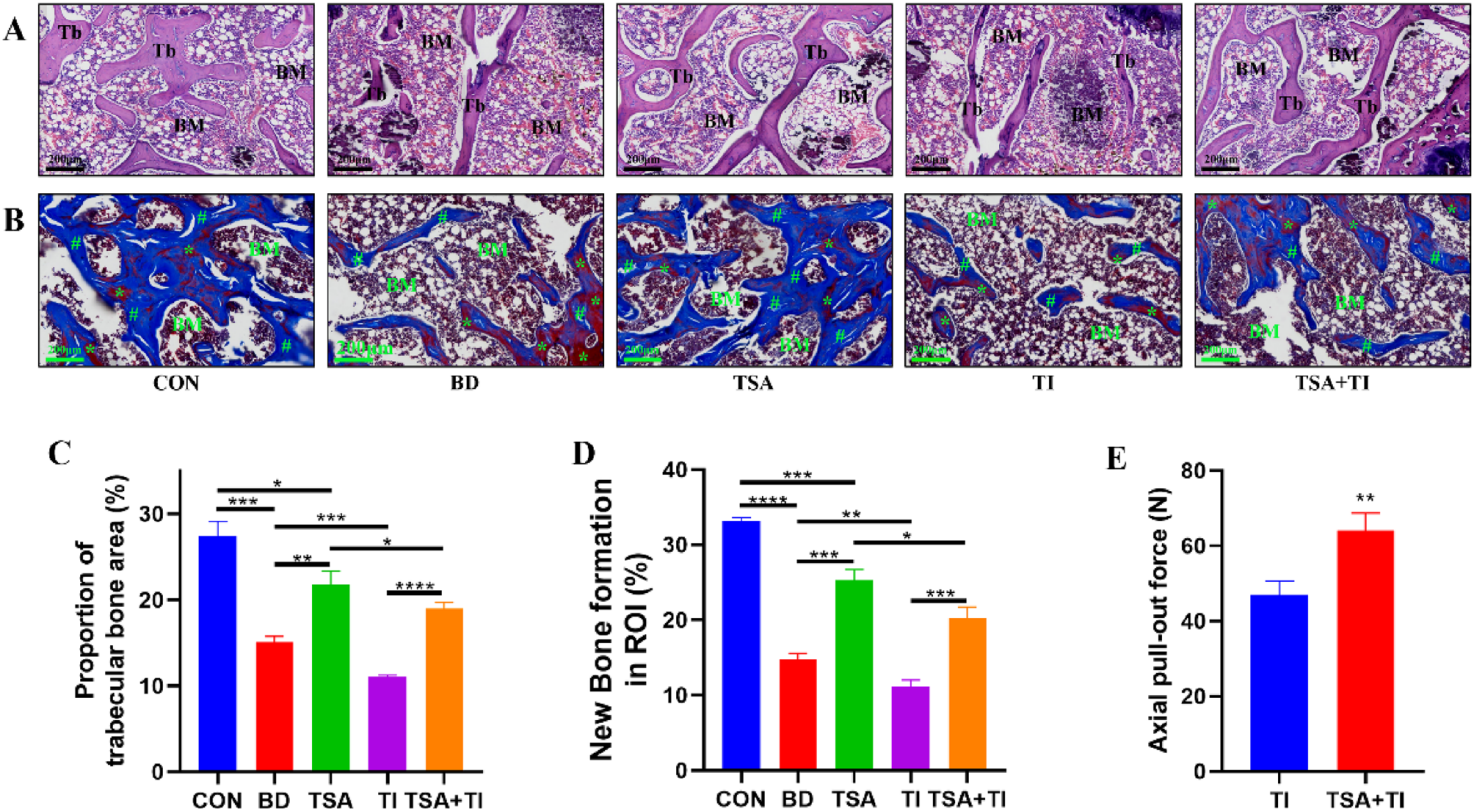
Results of HE and Masson's trichrome staining. (**A**) HE staining images at femur metaphysis. Scale bar = 200 μm; (**B**) Masson's trichrome staining images at femur metaphysis. Scale bar = 200 μm. (**C**) Proportion of trabecular bone area in ROI. (**D**) Histogram of new bone formation rate; (**E**) Axial pull-out force of titanium rods. Tb: trabecular bone. BM: bone marrow. Asterisk: mature bone. Pound sign: new bone and collagen fibers (*P < 0.05, **P < 0.01, ***P < 0.001, ****P < 0.0001).

Peak axial pull-out force was significantly higher in the TSA + TI group (64.20 ± 13.47 N) than in the TI group (46.98 ± 8.92 N) (Fig. 9E).

### Results of H&E and Masson's trichrome staining

H&E staining results (Fig. 9A,C) reveal that metaphyseal trabeculae in the CON group were dense and continuous. In contrast, the BD group displayed a reduction in trabecular numbers and the presence of fractures. The TSA group showed an increase in trabecular numbers compared to the BD group, although less than the CON group. Masson's trichrome staining (Fig. 9B,D) demonstrated higher levels of both new and mature bone formation in the CON group compared to the BD group. TSA treatment further augmented new bone formation, with the TSA + TI group exhibiting significantly higher new bone levels than the TI group.

### Results of immunohistochemistry staining

**Figure 10:**
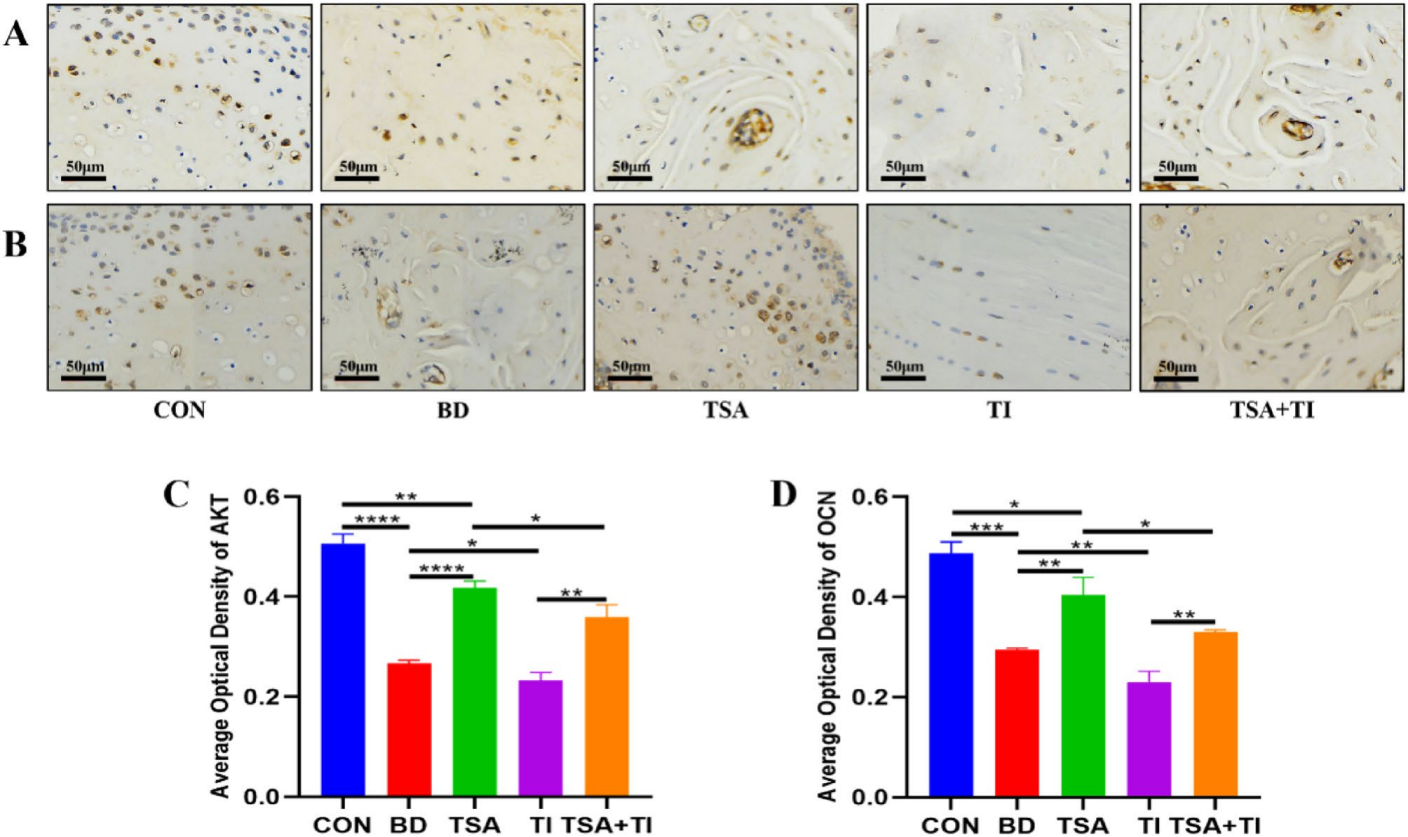
Results of immunohistochemistry staining. (**A**) Results of AKT immunohistochemistry staining, scale bar = 50 μm; (**B**) Results of OCN immunohistochemistry staining, scale bar = 50 μm; (**C**,**D**) Immunohistochemical quantitative analysis of the differences in AKT and OCN expression among the five groups (*P < 0.05, **P < 0.01, ***P < 0.001, ****P < 0.0001).

Immunohistochemical analysis revealed that the expression levels of both AKT and OCN decreased in the BD group compared to the CON group (Fig. 10A,B). In contrast, the TSA group exhibited increased expression levels of these proteins relative to the BD group. While the TI group displayed a slight reduction in these protein levels, the TSA + TI group demonstrated a significant increase in expression compared to the TI group alone. Quantitative analysis (Fig. 10C,D) further confirmed the elevated expression of AKT and OCN in the TSA group compared to the BD group, with the TSA + TI group showing higher levels than the TI group.

### BMSCs osteogenic induction assays

**Figure 11:**
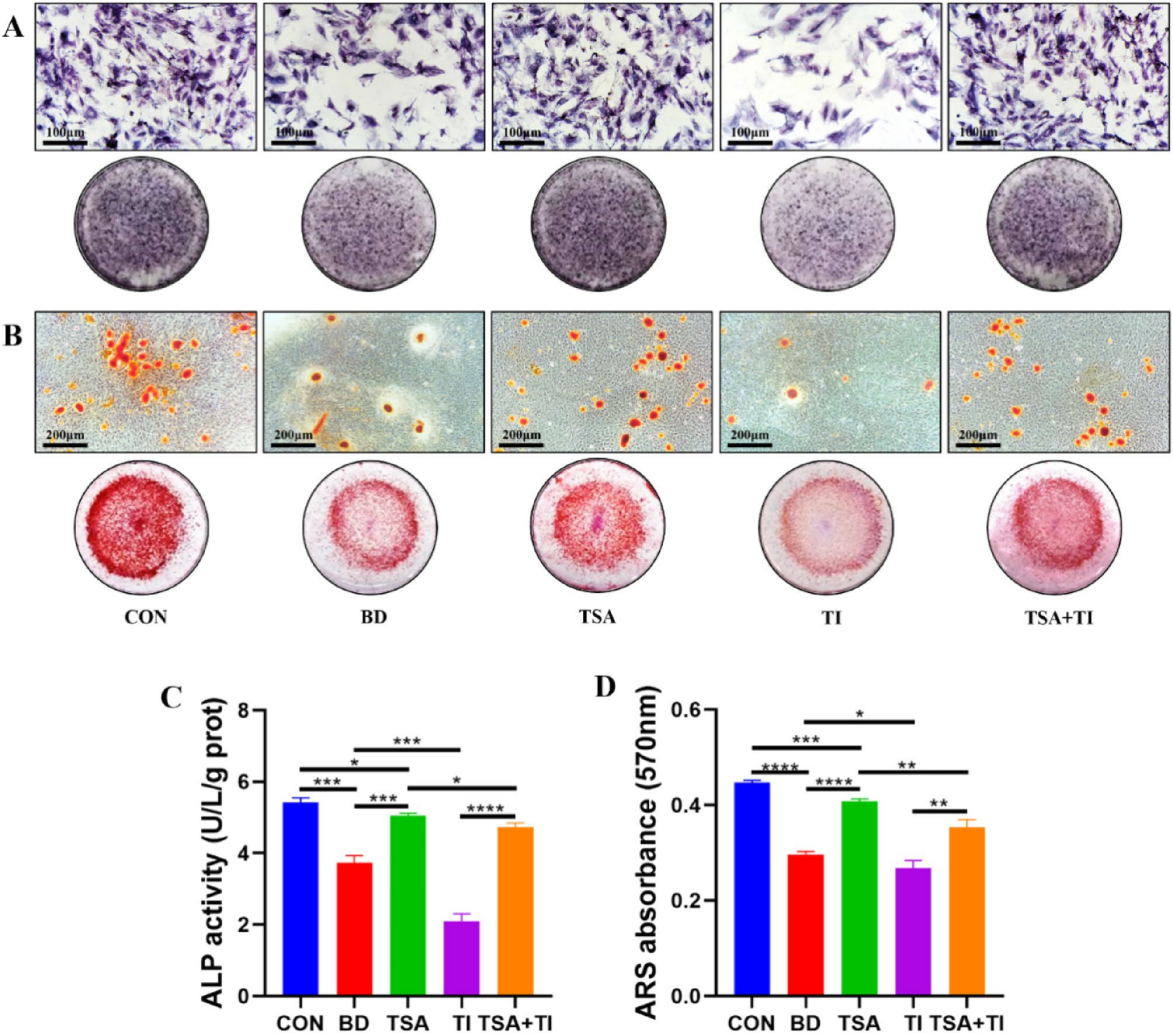
Effects of different treatments on alkaline phosphatase and osteogenic mineralization function of rat BMSCs. (**A**) Results of ALP staining, scale bar = 100 μm; (**B**) Results of ARS staining, scale bar = 200 μm; (**C**) ALP activity diagram; (**D**) Absorbance histogram of ARS at 570 nm (*P < 0.05, **P < 0.01, ***P < 0.001, ****P < 0.0001).

BMSCs isolated from the various treatment groups underwent ALP and ARS staining on days 7 and 21 post-osteogenic induction. The CON group cells exhibited a darker blue and purple hue (Fig. 11A,C), with a notable increase in calcified nodule formation (Fig. 11B,D), indicative of enhanced osteogenic differentiation and mineralization. In contrast, the BD group showed a significant reduction in these nodules. The TSA group cells demonstrated a marked increase in both nodule quantity and staining intensity. When compared to the TI group, the TSA + TI group cells had more calcified nodules and displayed more intense staining, suggesting improved osteogenic activity.

## Discussion

The PI3K/AKT pathway has been established as an essential regulator of osteogenesis and bone tissue growth^28^, with signaling through this pathway promoting the pronounced upregulation of osteogenic genes^29^. In contrast, the PI3K inhibitor LY294002 has been demonstrated to counteract these osteogenic effects, reversing the enhanced bone tissue growth and gene upregulation induced by the pathway^30,31^. The stimulation of PI3K leads to the transfer of AKT to the cellular membrane and its phosphorylation at Thr308/Ser473, after which it can regulate a range of physiological processes, including glucose metabolism and protein synthesis, thus altering overall cell function^32^. The Kelch-like Epichlorohydrin-related protein 1 (Keap1)/Nrf2/Antioxidant response element (ARE) antioxidant pathway, which is closely tied to oxidative stress incidence within cells, functions downstream of PI3K/AKT signaling^33^. This signaling pathway is integral to several cellular processes, encompassing apoptosis, cell cycle regulation, autophagy, and stem cell differentiation. Furthermore, aberrations in this axis have associations with various inflammatory and neurodegenerative diseases^34,35^.

**Figure 12:**
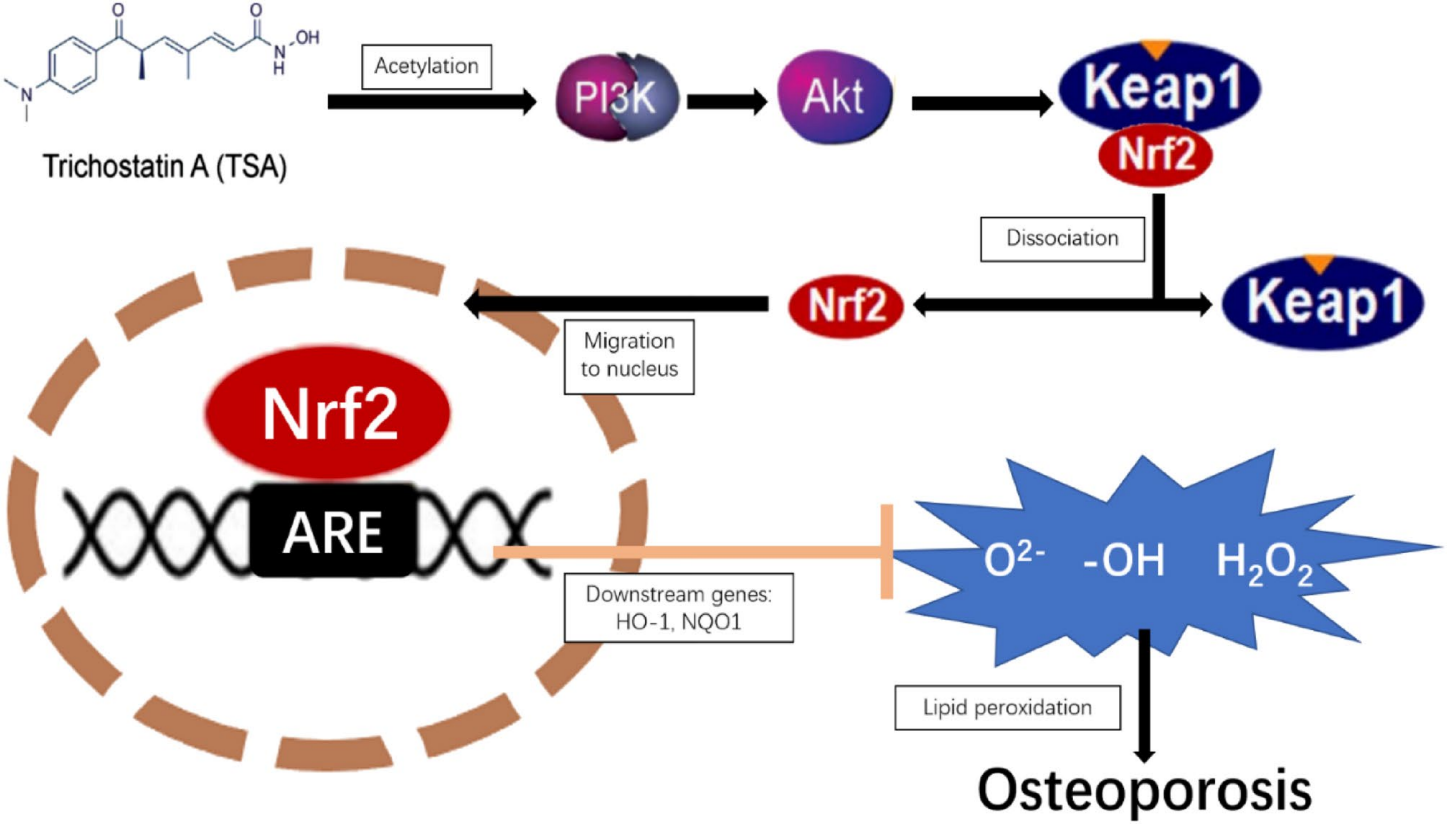
Mechanism of TSA activation of PI3K/AKT/Nrf2 pathway.

Recent studies have shown that low concentrations of TSA activate PI3K/AKT signaling^36,37^. As such, TSA may be capable of inhibiting the oxidative stress-induced apoptotic death of osteoblasts via the PI3K/AKT/Nrf2 signaling axis, thereby enhancing bone regeneration and the formation of new bone tissue. In alignment with these findings, TSA treatment triggers cellular acetylation, activating PI3K/AKT signaling and stimulating the upregulation of genes responsible for cellular protection (Fig. 12). At the molecular level, AKT induces the dissociation of Nrf2 from Keap1 in the cytosol, allowing Nrf2 into the nuclear compartment^38^. In the nucleus, Nrf2 and ARE interact and promote the upregulation of protective antioxidant genes, including HO-1, NQO1, Glutamate Cysteine ligase, Glutathione S-transferase, and Peroxiredoxin 1. By reducing intracellular ROS levels, the proteins encoded by these genes help maintain the redox balance, which is essential for protecting cells and tissues from oxidative damage^39^. Prior reports have emphasized the key role that excess ROS production plays in OP pathogenesis^40,41^. The main components of ROS include O_2_^−^, –OH, and H_2_O_2_^42^. The H+ ionophore CCCP can readily uncouple mitochondrial oxidative phosphorylation, increasing inner mitochondrial membrane H+ permeability such that the MMP across the inner mitochondrial membrane decreases, inducing apoptotic death^43,44^. CCCP exposure thereby induces the production of O_2_^−^, –OH, and limited amounts of H2O2, mimicking exposure to persistent oxidative stress. Compared to traditional H2O2-mediated models, CCCP-mediated oxidative stress provides a more stable and controllable method for inducing cellular responses, enhancing the reliability of experimental outcomes^45,46^. Decreased MMP can promote ROS generation, thereby promoting mitochondrial cytochrome C release and caspase-3 activation^47,48^. When ROS production is excessive, lipid peroxidation can occur, and damage can be sustained by a range of macromolecules, including lipids, proteins, and nucleic acids^49,50^. Lipid peroxidation byproducts can drive apoptotic cell death by regulating NF-κB, MAPK, and PKC pathway activity^51^. Overly high ROS levels can also consume available antioxidant reserves of SOD, catalase, and glutathione peroxidase within cells, thereby reducing overall antioxidant capacity in these cells^52^. CCCP treatment caused noticeable swelling and rounding of MC3T3-E1 cells, along with increased intracellular ROS levels and decreased MMP, indicative of impaired cellular activity and progression toward apoptosis. CCCP treatment thus successfully drove oxidative stress injury to these MC3T3-E1 cells. In line with these findings, measurements of oxidative stress detection indices demonstrated the ability of TSA treatment to alleviate oxidative damage in MC3T3-E1 cells, as evidenced by reduced MDA content, decreased ROS generation, and higher levels of SOD activity. TSA was also sufficient to decrease the ratio of cells in the early and late stages of apoptosis as detected via flow cytometry while further increasing levels of key osteogenesis-associated proteins, including OPN, OCN, Runx2, and BMP-2. This coincided with cleaved caspase-3 and Bax levels reductions and higher total caspase-3 and Bax expression. These findings indicate that TSA can suppress oxidative stress, enhance mitochondrial function, and stimulate both bone regeneration and new bone formation. TSA treatment also increased levels of AKT phosphorylation within these MC3T3-E1 cells. Furthermore, subcellular fractionation assays showed that TSA increased nuclear and overall Nrf2 levels while reducing cytosolic Nrf2 levels. This pattern is consistent with TSA's role in facilitating Nrf2 dissociation from Keap1, promoting its nuclear entry, and subsequently enhancing the upregulation of NQO1 and HO-1. The co-treatment of cells with the PI3K inhibitor LY294002 was sufficient to partially reverse the effects of TSA on these cells, as evidenced by reductions in AKT expression, lower levels of total and nuclear Nrf2, and the suppression of NQO1 and HO-1 expression. TSA can thus alleviate oxidative stress, promote new bone formation, facilitate bone regeneration, and enhance osseointegration, at least in part through inducing PI3K/AKT/Nrf2 signaling. MC3T3-E1 cells were further treated with nanoscale titanium powder to simulate titanium implant exposure in the context of OP in vitro. ALP staining revealed higher levels of titanium particles within the cytosol of cells treated using TSA consistent with TSA having enhanced titanium fusion with these target cells. TSA treatment resulted in a more pronounced purple and blue staining, a change that indicates enhanced osteogenic mineralization. The results of Western blot further demonstrated that titanium powder did not impact the ability of TSA to promote osteogenesis-associated protein upregulation or to protect against ROS generation or apoptotic death in these MC3T3-E1 cells.

Subsequent in vivo experiments were conducted to validate these conclusions. The OVX rat model of OP used for this study is commonly employed for studying the OP mechanisms in vivo^53,54^. The active homeostatic balance between the resorption and formation of bone tissue is central to maintaining a healthy skeletal system^55^. When bone is resorbed at a rate faster than new bone can be produced, net bone loss occurs, which drives OP development^56^. These experiments showed a reduction in BMD in the OVX group compared to the sham control group, with H&E staining further uncovering distinct structural abnormalities in the OVX specimens. These results confirmed the successful establishment of this OVX-induced OP model, enabling the further use of these rats to simulate fractures with a bone defect model followed by titanium rod implantation. Micro-CT scans, complemented by 3D reconstruction, monitored bone formation in these rat treatment groups. Separately, we assessed the force required to extract titanium rods from the bone, maintaining a constant speed during the extraction process. Trabecular bone quantity, spacing, and thickness were assessed through H&E staining, while new and mature bone levels in the target ROI were detected via Masson's staining. Immunohistochemical staining uses a specific binding reaction between an antigen and an antibody to color and display a protein of interest in a cell or tissue in blue. So, immunohistochemical assays were conducted to confirm that the TSA mechanisms observed in rats align with those identified in vitro. At the same time, BMSCs were harvested to directly investigate the osteogenic mineralizing activity of primary osteoblasts. Overall, these experiments showed significant improvements in bone healing and new bone formation with TSA treatment, underscoring its efficacy in promoting osteogenesis in the OP model. Being foreign bodies, titanium implants potentially disrupt bone healing, contributing to friction, aseptic inflammation, and loosening^57,58^. This phenomenon correlates with the diminished trabecular bone integrity observed in the TI group compared to the BD group. Following treatment with TSA, however, trabecular bone and new bone development surrounding implanted titanium rods were significantly augmented. The immunohistochemical results showed that the mechanism of action of TSA in rats was consistent with that of in vitro experiments. TSA enhanced the expression level of AKT in rats, indicating that TSA promoted bone healing and osseointegration in rats, at least in part by activating the AKT/Nrf2 signaling pathway. We observed increased ALP activity in BMSCs from TSA-treated rats post-osteogenic differentiation. Additionally, ARS staining revealed more calcified nodules, indicating elevated osteogenic mineralization capacity in these cells. Relative to the TI group, significant improvements in titanium rod pull-out resistance were also observed in the TSA + TI group, consistent with the greater integration of these implants into the surrounding bone. Overall, these results highlight the ability of TSA to promote the formation of new bone tissue and to enhance titanium rod integration into the surrounding bone in an OVX-induced rat model of OP.

TSA is thus capable of promoting the formation of new bone and enhancing osseointegration in an OVX-induced rat model of OP via PI3K/AKT/Nrf2 pathway activation. Through a series of in vitro and in vivo experiments, our analyses elucidate the mechanisms by which TSA treatment facilitates the osseointegration of titanium rods in an OVX rat model of OP. However, there are certain limitations. First, no bone parameters in regions beyond the ROI were analyzed, and it thus remains to be determined whether TSA affects bone tissue locally or throughout the body. Additionally, the processes underlying osseointegration at the micro-scale were not studied in these experiments, providing a clear direction for follow-up research.

## Conclusions

In conclusion, our findings underscore the novel role of TSA in stimulating the AKT/Nrf2 pathway, thereby mitigating oxidative stress-induced OP and enhancing titanium implant integration. These results suggest that TSA treatment could be a promising therapeutic strategy for managing OP-related fractures.

### Supplementary Information

Supplementary Information.

## Data Availability

The datasets used and/or analyzed during the current study are available from the corresponding author upon reasonable request.
