# Supplementary material for: Trichostatin A enhances the titanium rods osseointegration in osteoporotic rats by the inhibition of oxidative stress through activating the AKT/Nrf2 pathway
Source: Sci Rep. 2023 Dec 27;13:22967. doi: 10.1038/s41598-023-50108-1 (PMC10752907; doi:10.1038/s41598-023-50108-1)

Trichostatin A enhances the titanium rods osseointegration in osteoporotic rats by the inhibition of oxidative stress through activating AKT/Nrf2 pathway

Zhi Zhou[#], Wenkai Jiang[#], Junjie Yan, Hedong Liu, Maoxian Ren, Yang Li, Zhiyi Liu, Xuewei Yao, Tianlin Li, Nengfeng Ma, Bing Chen, Wengang Guan, Min Yang*

Department of Traumatology and Orthopedics, Yijishan Hospital, Wannan Medial College, Wuhu Anhui, 241001, P.R.China

*Corresponding author: Min Yang (pkuyang@hotmail.com)

[#]Zhi Zhou and Wen-kai Jiang have contributed equally to this work

Original Blots

Due to the large workload of our western blotting and the limited funding, blots are cut prior to hybridization with antibodies to economize PVDF membranes. We cut out the band where our target protein is located according to the color and spacing of the marker, and then mark bands with a ballpoint pen (protein types, front and back). The cropped bands include at least two markers above and below the target protein (but in practice, sometimes the marker is not clear). Below is the original blots merge image (exposure plot and normal image) of western blotting in our study.

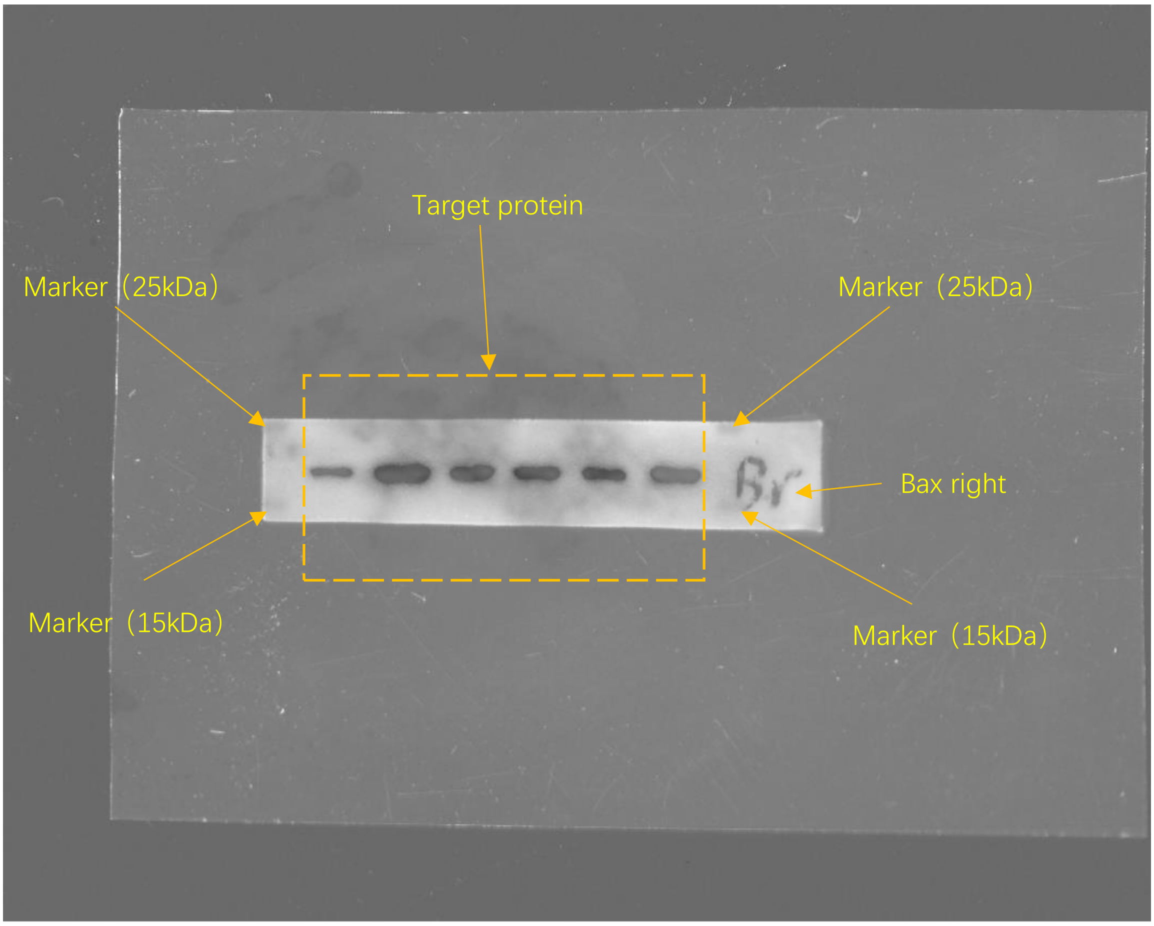

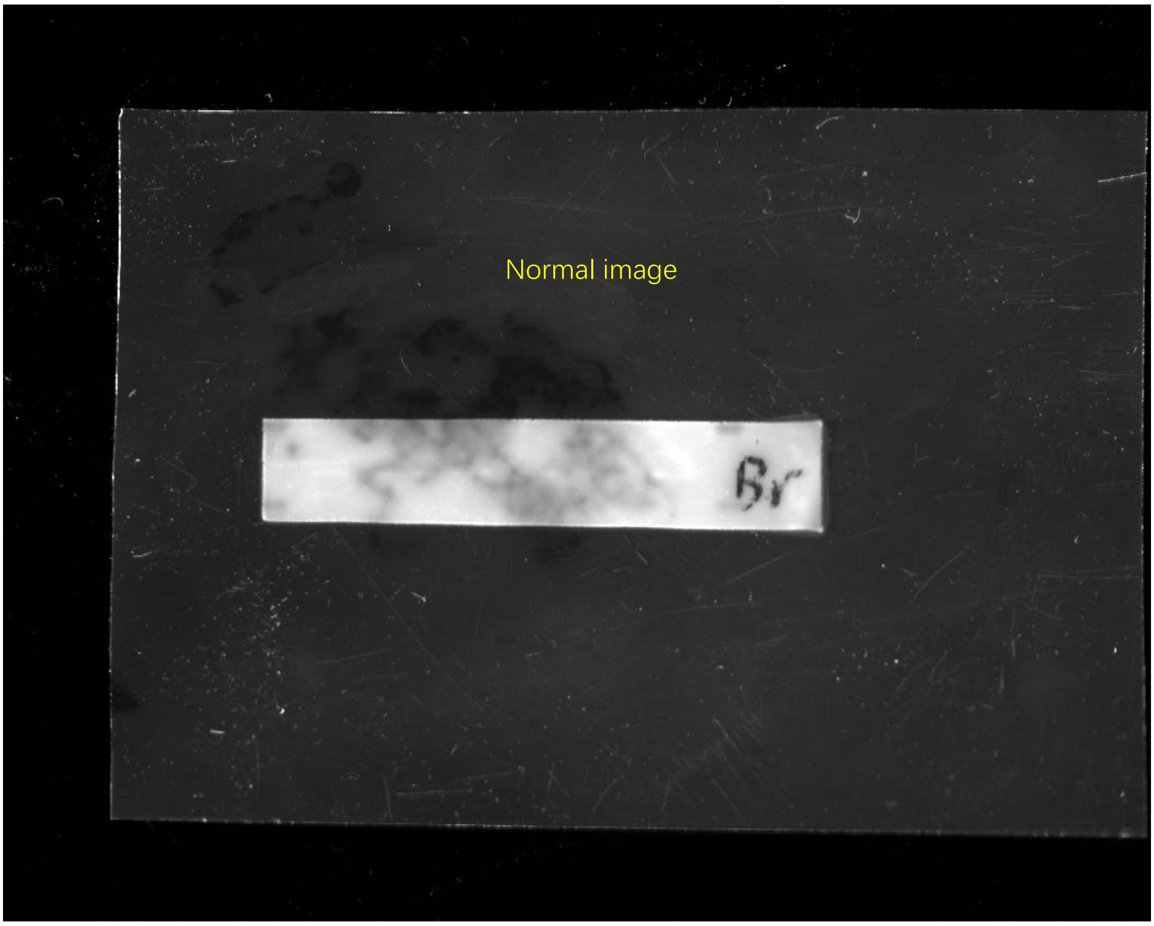
Normal image
Br

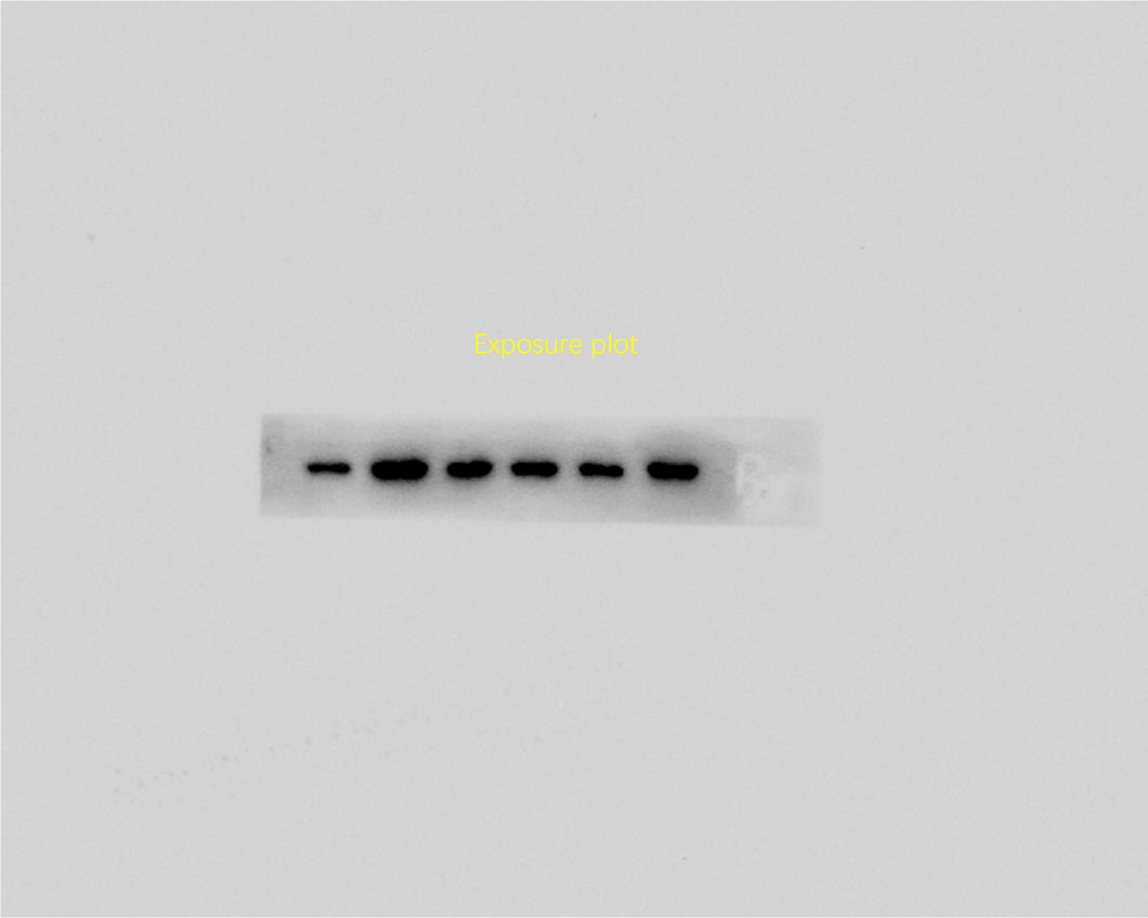
Exposure plot

Supplementary Panel A.

Display of original blots for OPN, Runx2, BMP2, OCN, Caspase3, Bcl2, Cleaved Caspase3, AKT, HO-1, NQO1 and β-actin Western blot analysis (Fig. 4A, B).

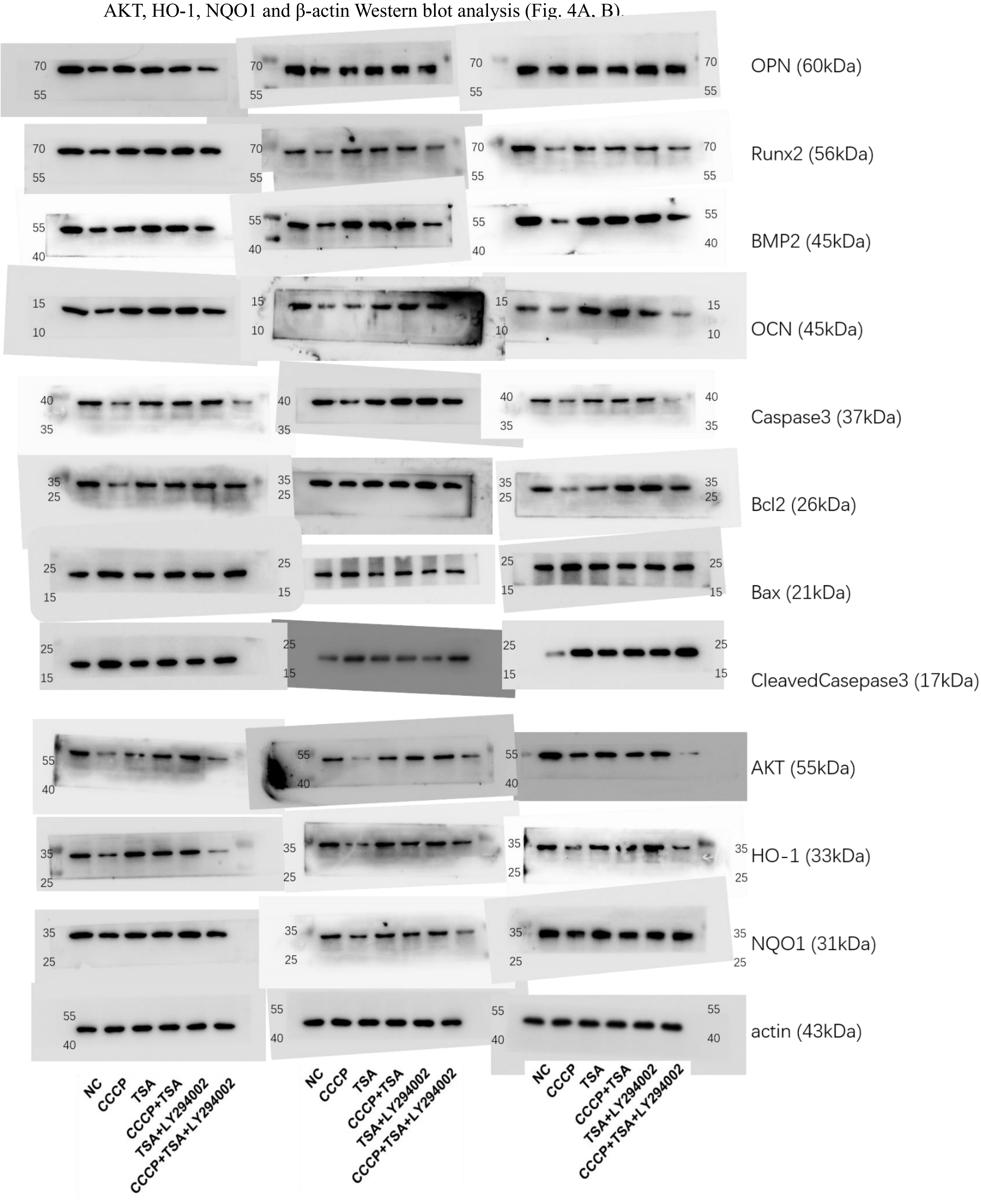

Supplementary Panel B.

Display of original blots for Nuclear Nrf2, Histone 3, Cytosol Nrf2, Total Nrf2 and β-actin Western blot analysis (Fig. 5A, B).

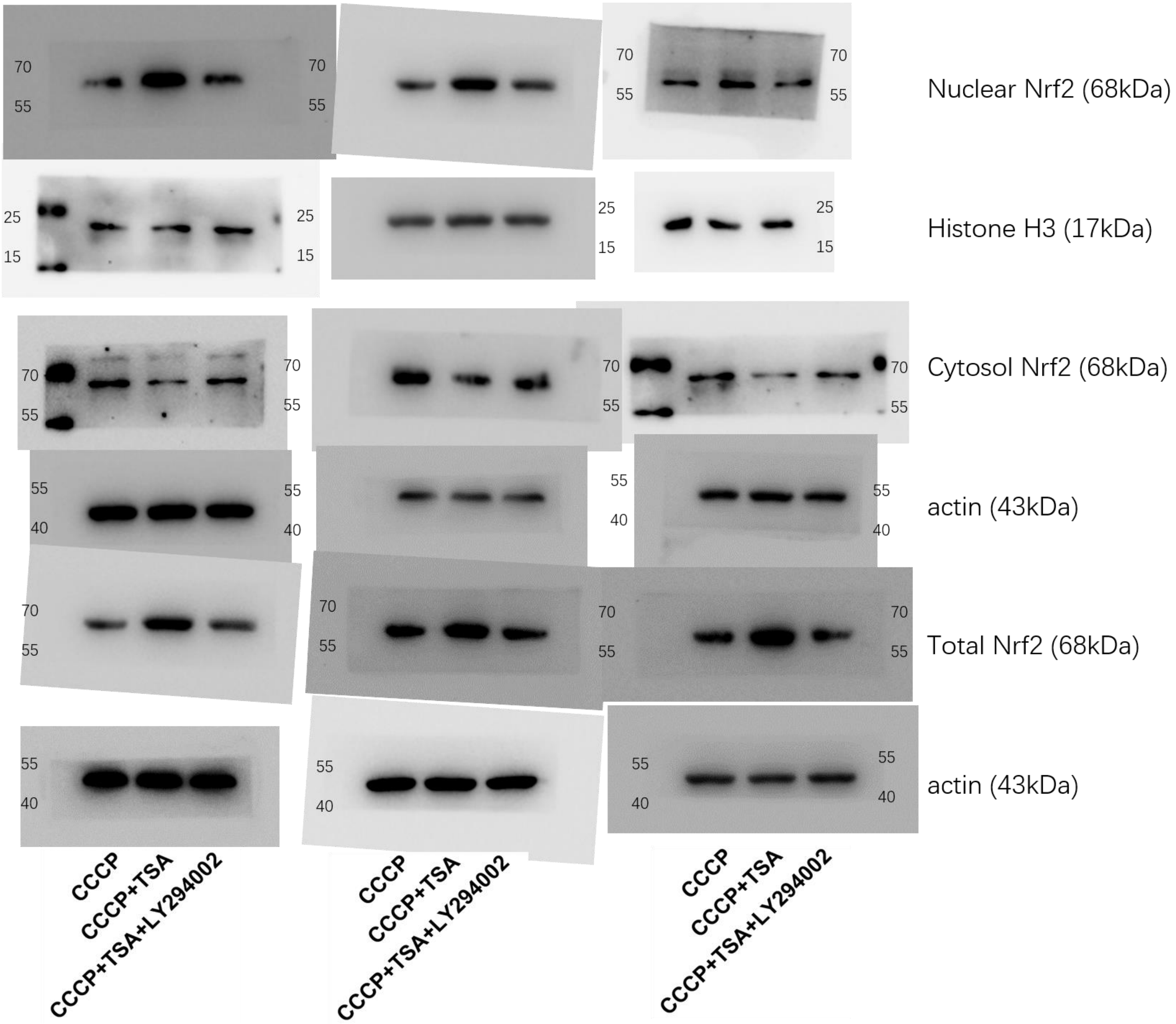

Supplementary Panel C.

Display of original blots for OPN, Runx2, BMP2, OCN, Caspase3, Bcl2, Cleaved Caspase3, AKT, HO-1, NQO1 and β-actin Western blot analysis (Fig. 5C, D).

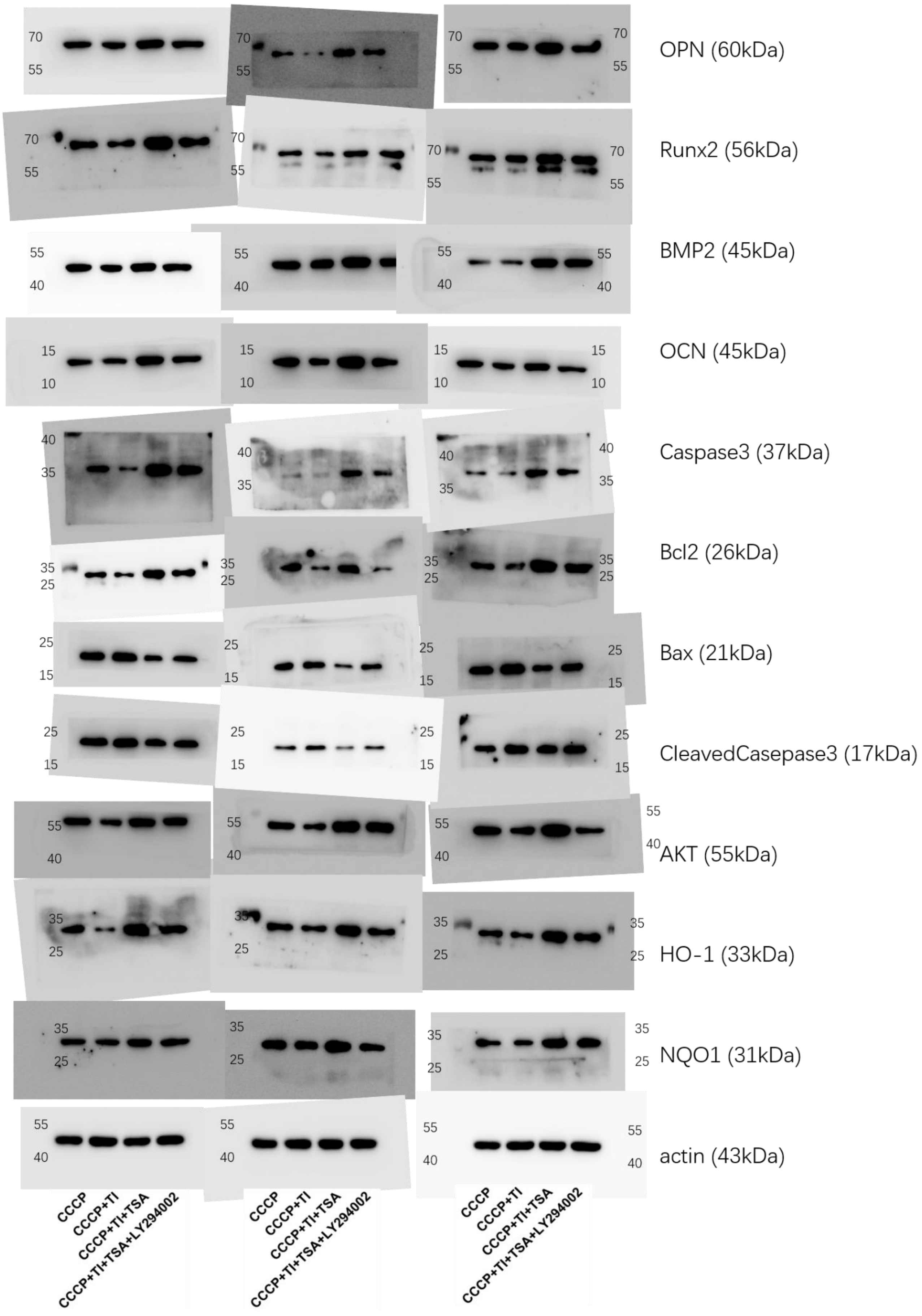

Supplement: Supplementary file 1 — Supplementary Information. [file 41598_2023_50108_MOESM1_ESM.pdf]
